# Quantitative Ultrastructural Morphometry and Gene Expression of mTOR-Related Mitochondriogenesis within Glioblastoma Cells

**DOI:** 10.3390/ijms21134570

**Published:** 2020-06-27

**Authors:** Rosangela Ferese, Paola Lenzi, Federica Fulceri, Francesca Biagioni, Cinzia Fabrizi, Stefano Gambardella, Pietro Familiari, Alessandro Frati, Fiona Limanaqi, Francesco Fornai

**Affiliations:** 1 I.R.C.C.S. Neuromed, via Atinense 18, 86077 Pozzilli (IS), Italy; ferese.rosangela@gmail.com (R.F.); francesca.biagioni@neuromed.it (F.B.); stefano.gambardella@neuromed.it (S.G.); alessandro.frati@uniroma1.it (A.F.); 2 Department of Translational Research and New Technologies in Medicine and Surgery, University of Pisa, via Roma 55, 56126 Pisa, Italy; paola.lenzi@unipi.it (P.L.); f.limanaqi@studenti.unipi.it (F.L.); 3 Department of Clinical and Experimental Medicine University of Pisa, via Roma 55, 56126 Pisa, Italy; federica.fulceri@unipi.it; 4 Department of Anatomy, Histology, Forensic Medicine and Orthopedics, Sapienza University of Rome, Via A. Borelli 50, 00161 Rome, Italy; cinzia.fabrizi@uniroma1.it; 5 Department of Biomolecular Sciences, University of Urbino “Carlo Bo”, 61029 Urbino, Italy; 6 Department of Human Neurosciences, Division of Neurosurgery, Sapienza University of Rome, 00185 Roma, Italy; pietro.familiari@uniroma1.it

**Keywords:** autophagy, mitophagy, lysosomes, mitochondrial biogenesis, mitochondrial DNA, PGC1α, NRF2, mitochondrial constitutive genes, rapamycin

## Abstract

In glioblastoma (GBM) cells, an impairment of mitochondrial activity along with autophagy suppression occurs. Autophagy suppression in GBM promotes stemness, invasion, and poor prognosis. The autophagy deficit seems to be due, at least in part, to an abnormal up-regulation of the mammalian target of rapamycin (mTOR), which may be counteracted by pharmacological mTORC1 inhibition. Since autophagy activation is tightly bound to increased mitochondriogenesis, a defect in the synthesis of novel mitochondria is expected to occur in GBM cells. In an effort to measure a baseline deficit in mitochondria and promote mitochondriogenesis, the present study used two different GBM cell lines, both featuring mTOR hyperactivity. mTORC1 inhibition increases the expression of genes and proteins related to autophagy, mitophagy, and mitochondriogenesis. Autophagy activation was counted by RT-PCR of autophagy genes, LC3- immune-fluorescent puncta and immune-gold, as well as specific mitophagy-dependent BNIP3 stoichiometric increase in situ, within mitochondria. The activation of autophagy-related molecules and organelles after rapamycin exposure occurs concomitantly with progression of autophagosomes towards lysosomes. Remarkably, mitochondrial biogenesis and plasticity (increased mitochondrial number, integrity, and density as well as decreased mitochondrial area) was long- lasting for weeks following rapamycin withdrawal.

## 1. Introduction

Defective autophagy characterizes astrocyte-derived brain tumours, and mostly glioblastoma multiforme (GBM) [1,2,3,4,5,6]. This depends on a robust up-regulation and over-activity of the mammalian target of rapamycin complex I (mTORC1) [7,8,9,10,11]. In fact, in patients and experimental models there is a clear correlation between mTOR over-activity, autophagy depression and tumour malignancy [12,13,14,15,16,17,18,19,20,21,22,23]. The ability of mTORC1 inhibitors to rescue autophagy in glioblastoma was shown in vivo and in vitro extending from cell lines to patients’ cell cultures and in vivo xenografts [6,24,25]. 

When autophagy is rescued, beneficial effects are produced within GBM cells [6,18,19,21,26,27,28,29,30]. At the same time, autophagy activation dose-dependently produces cell differentiation and a loss of stemness [21]. This happens when rapamycin is administered at doses falling within its therapeutic range (13–22 nM) and reaching a plateau at 10 nM [31,32,33,34,35]. Apart from empowering the clearance of protein cargoes, autophagy eventually alters the mitochondrial status (mitophagy), which makes it unlikely to stimulate autophagy without producing a concomitant mitochondrial remodelling [36,37,38]. This becomes a key point when considering the neurobiology of GBM, where mitochondrial status is aberrantly reduced [39,40,41,42]. In fact, impairment of mitochondrial activity characterizes GBM, where cell metabolism is shifted towards anaerobiosis [40,42]. The biogenesis of mitochondria and activation of autophagy and mitophagy machinery are tightly connected when considering protein interaction and gene expression. Therefore, in the present study we selected an effective dose of rapamycin at different time intervals to measure various outcomes such as: autophagy vacuoles, autophagy protein and genes, as well as autophagy progression combined with mitophagy and mitochondriogenesis by using a number of experimental techniques. This approach allows expressions of various genes and proteins to be quantified which have been recruited during autophagy- related mitochondriogenesis while comparing these data with changes in mitochondrial DNA, genes, number, and phenotype. To further validate these data, we replicated all the experiments in two different GBM cell lines. In detail, we analysed the ultrastructural morphometry of mitochondria and autophagy structures as well as the merging with lysosomes with transmission electron microscopy (TEM) at different time intervals (ranging from 1 d up to 14 d following rapamycin withdrawal). The analysis was carried out following different doses (from 1 nM up to 100 nM) and times (12 h and 24 h) of continuous rapamycin administration, considering the therapeutic range of rapamycin (13–22 nM) in humans [21,35]. The time intervals needed to be specified considering that rapamycin was administered for 12 h or 24 h, then rapamycin was withdrawn for 24 h up to 14 d. This means that the first time interval of rapamycin withdrawal is indicated as 1 d and it corresponds to rapamycin administration for 24 h followed by 24 h of withdrawal. All later time intervals refer to the amount of time of withdrawal. In the very same samples, we quantified stoichiometry of site-specific placement and amount of autophagy- and mitophagy-related molecules with an emphasis on those biochemical pathways bridging autophagy and mitochondriogenesis and autophagosomes merging with lysosomes [36,37,38,43]. In fact, rapamycin-dependent modulation of mitochondriogenesis within GBM cells is concomitant with activation of autophagy, mitophagy, and autophagy progression towards lysosomes. Specific quantitative assessment of mitochondriogenesis, autophagy structures, autophagy progression, and mitophagy was carried out in the same experimental settings. In fact, the present experiments provide the measure of autophagy markers both at gene, protein, and morphological level, along with measurement of mitochondrial biogenesis (again assessed at gene, protein, and morphological level) including in situ detection of key proteins within mitochondria. A quantitative calculation of genes expressions related with mitochondriogenesis and authentic mitochondrial genes was carried out. The downregulation of mTORC1 achieved by rapamycin in the present study is supported by semi-quantitative-analyses based on immune-blotting showing a reduced amount of mTORC1 downstream products. However, several additional studies are needed to complete our knowledge about the mitochondrial status in GBM while confirming the efficacy of rapamycin administration on mTORC1 activity suppression. This requires all these data to be replicated in vivo and dissected for each step through specific genetic manipulation. This can be achieved by overexpressing or silencing genes of mitochondriogenesis, or genes required to synthesize the mTORC1 complex as well as specific, step-dependent autophagy and mitophagy genes while measuring the efficacy of gene silencing in these experimental conditions. As a matter of fact, knockdown of *NRF2* blocks mitochondriogenesis [44,45,46]. Many more studies about genetic manipulation of lysosomal activity are needed. Our group is committed to this research activity for several years to come.

## 2. Results

### 2.1. Preliminary Experiments to Assess the Effects of Various Doses and Times of Rapamycin Administration on Mitochondrial Number in Different GBM Cell Lines 

**Figure 1:**
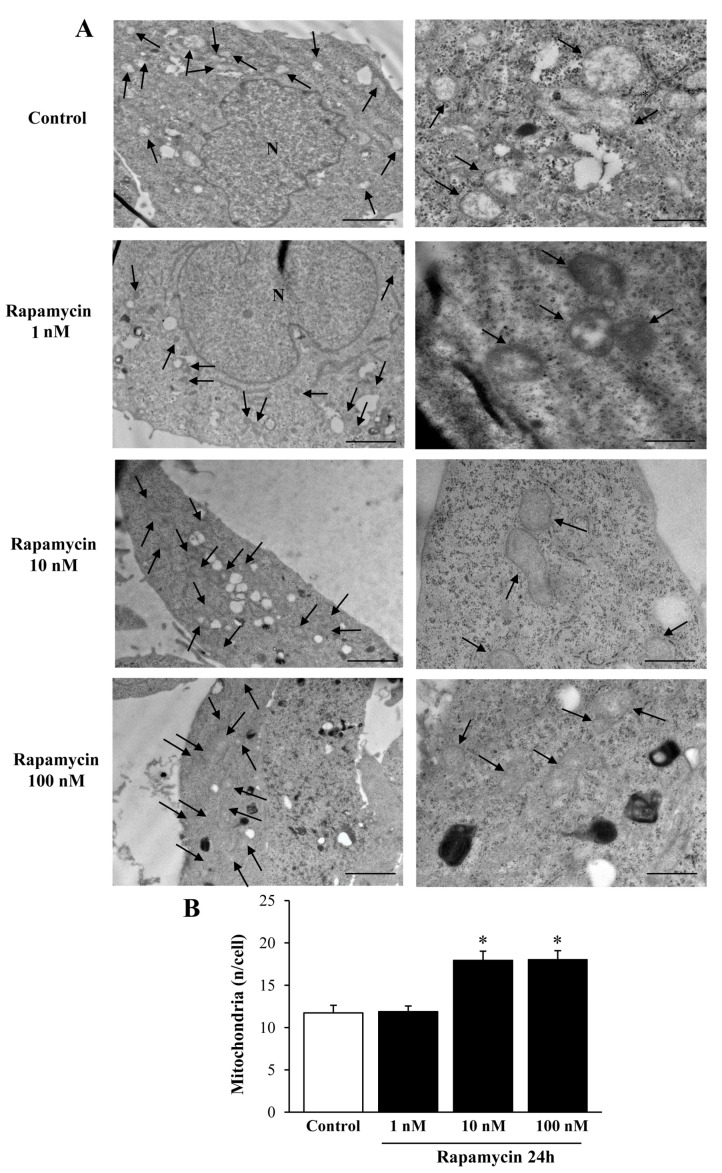
Rapamycin dose-dependently increases mitochondrial number in U87MG cell line. (**A**) Representative TEM micrographs showing mitochondria (indicated by black arrows) from Control and from different doses of rapamycin. (**B**) Graph reports the number of mitochondria per cell. Values are the mean ± S.E.M. from 50 cells per group. ∗ *p* ≤ 0.05 vs. Control and 1 nM rapamycin; Scale bars = 1 μm (low magnification) and 0.56 μm (high magnification).

**Figure 2:**
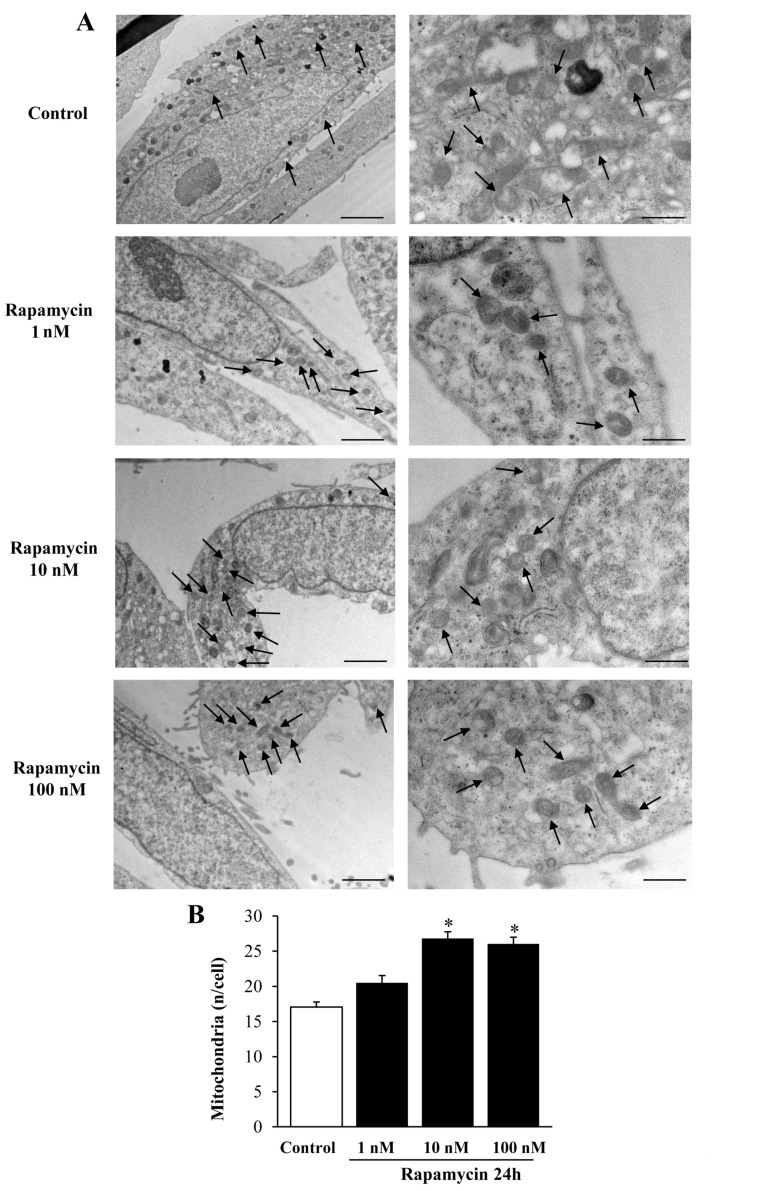
Rapamycin dose-dependently increases mitochondrial number in A172 cell line. (**A**) Representative TEM micrographs showing mitochondria (indicated by black arrows) from Control and from different doses of rapamycin. A more differentiated cell phenotype is evident in Control cells when compared to U87MG cells shown in Figure 1. (**B**) Graph reports the number of mitochondria per cell. Values are the mean ± S.E.M. from 30 cells per group. ∗ *p* ≤ 0.05 vs. Control and 1 nM rapamycin. Scale bars = 1 μm (low magnification) and 0.4 μm (high magnification).

**Figure 3:**
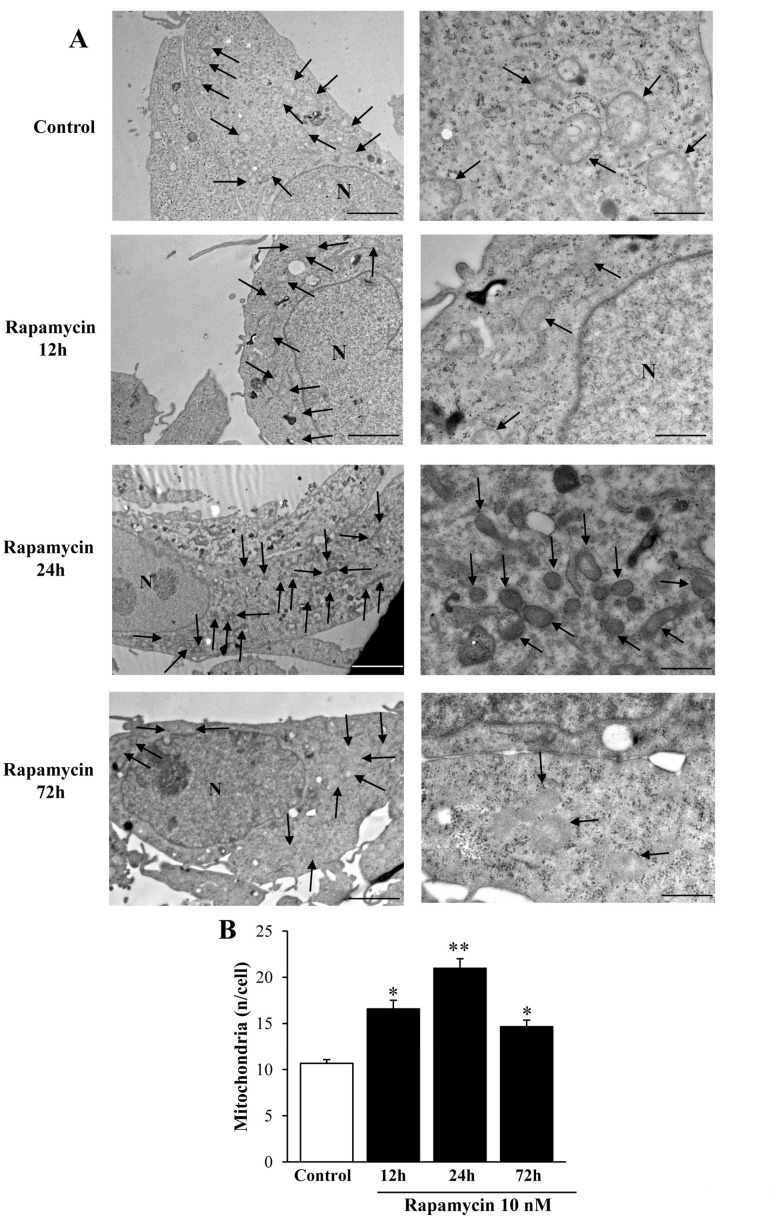
Rapamycin time-dependently increases mitochondrial number in U87MG cell line. (**A**) Representative TEM micrographs showing mitochondria (indicated by black arrows) from Control and from different time of continuous rapamycin 10 nM exposure. (**B**) Graph reports the number of mitochondria per cell. Values are the mean ± S.E.M. from 50 cells per group. ∗ *p* ≤ 0.05 vs. Control; ** *p* ≤ 0.05 vs. other groups. Scale bars = 1 μm (low magnification) and 0.56 μm (high magnification).

**Figure 4:**
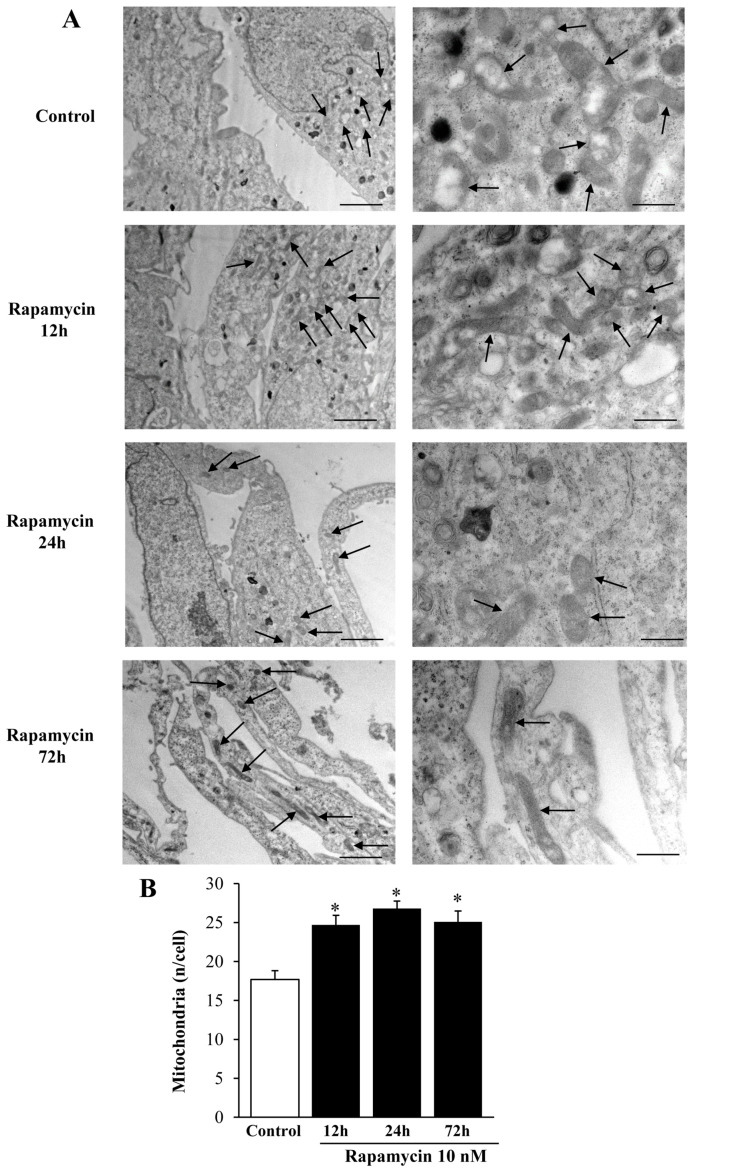
Rapamycin time-dependently increases mitochondrial number in A172 cell line. (**A**) Representative TEM micrographs showing mitochondria (indicated by black arrows) from Control and from different time of continuous rapamycin 10 nM exposure. (**B**) Graph reports the number of mitochondria per cell. Values are the mean ± S.E.M. from 30 cells per group. ∗ *p* ≤ 0.05 vs. Control. Scale bars = 1 μm (low magnification) and 0.45 μm (high magnification).

**Figure 5:**
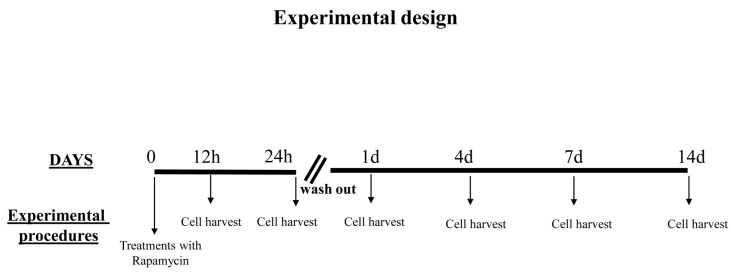
Overview of the experimental design. Rapamycin was administered continuously for 12 h or 24 h to the cell cultures; then, it was withdrawn for 24 h up to 14 d. Thus, the first time interval of rapamycin withdrawal (24 h) is indicated as 1 d and it corresponds to rapamycin administration for 24 h followed by 24 h of withdrawal. All later time intervals are reported considering the days the rapamycin withdrawal time lasted.

We measured the effects of various doses of rapamycin on the number of mitochondria in U87MG (Figure 1) and A172 (Figure 2) cell lines. The effects of rapamycin continuous exposure at various time intervals (12 h; 24 h; 72 h) were calculated on the number of mitochondria per cell as reported in Figure 3 and Figure 4 (U87MG and A172 cell lines, respectively). In both cell lines the dose of 10 nM rapamycin continuously administered for 12 h and mostly 24 h produced the highest mitochondrial number (Figure 1, Figure 2, Figure 3 and Figure 4). This is why in each experiment we selected this dose of 10 nM rapamycin, which was administered for 12 h and 24 h. However, only the 24 h, 10 nM rapamycin administration protocol was applied when long-lasting effects were measured at various time intervals: from 24 h up to 14 d following rapamycin withdrawal (as per experimental protocol reported in Figure 5). This is reported in the experimental design introducing the treatment protocols (Section 4.1). The two GBM cell lines used here provided similar results. Nonetheless, it should be considered that the cell phenotype was not fully overlapping. In fact, the A172 cell line features a greater cell size, and is more differentiated compared with the U87 MG cell line. We have already detailed these differences in a dedicated paper [21]. In the present study, we were able to add further discrepancies concerning the mitochondrial status. In fact, despite the number of mitochondria being lower in A172 cells, they were more abundant compared with the severe lack of mitochondria documented in U87MG cells. 

### 2.2. Rapamycin Produces a Two-Fold Increase in MitoTracker-Green (MTR-G) 

**Figure 6:**
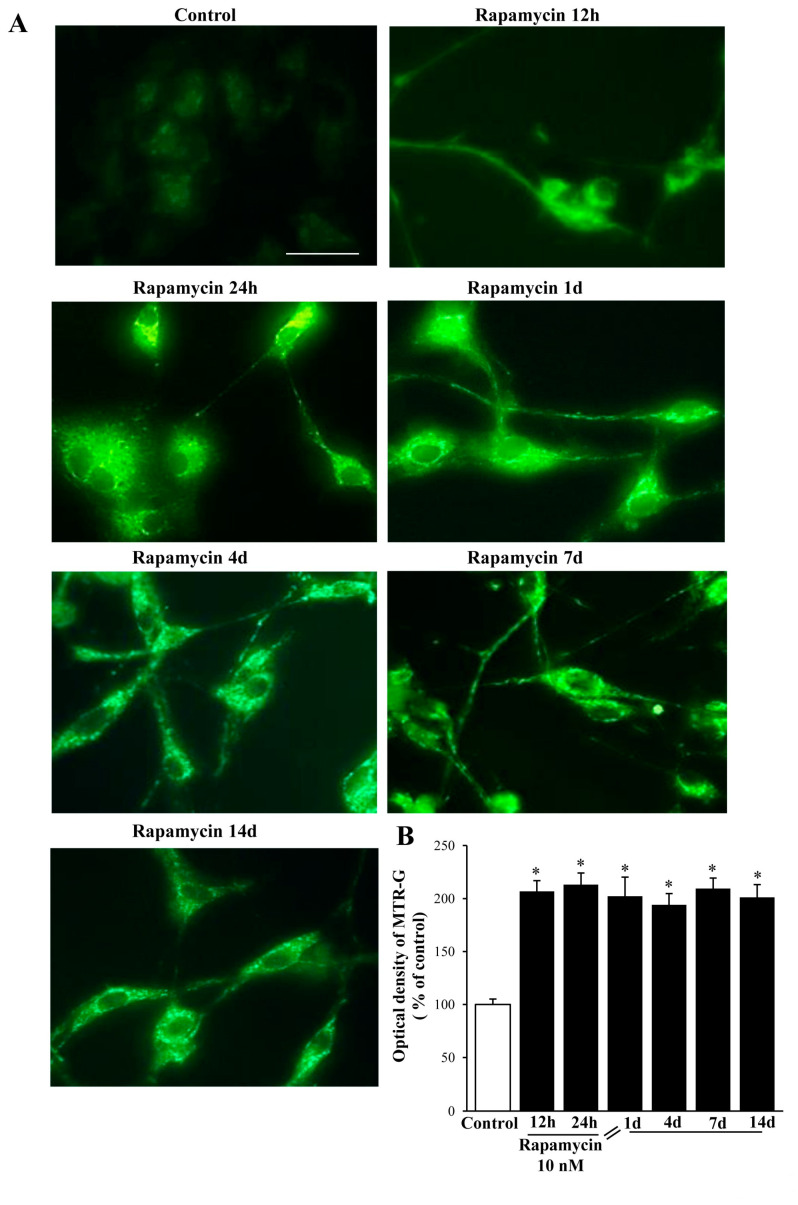
Rapamycin increases MitoTraker-Green (MTR-G) in U87MG cell line. (**A**) Representative fluorescent microscopy of U87MG cells stained with 250 nM of the mitochondrial MTR-G dye (green). (**B**) Graph reports fluorescence at different time intervals. Values are the mean percentage ± S.E.M. from 50 cells per group. * *p* ≤ 0.05 vs. Control. Scale bar = 25 μm.

**Figure 7:**
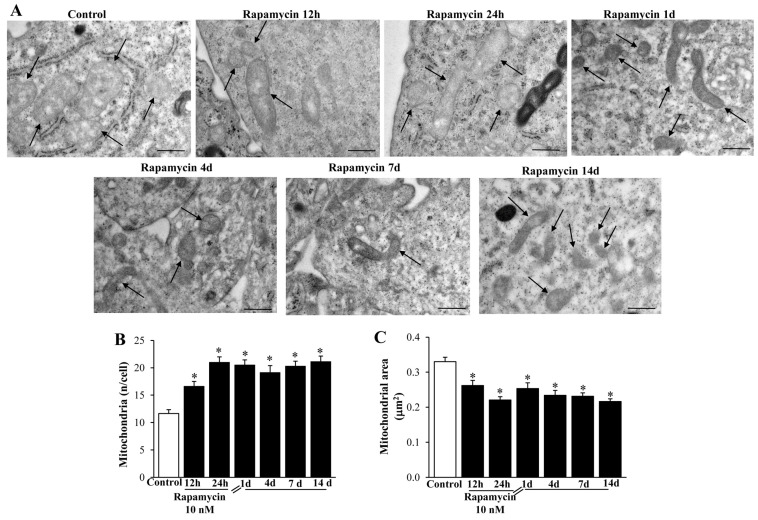
Rapamycin modifies mitochondrial number, area, and phenotype within U87MG cells. (**A**) Representative TEM micrographs showing mitochondria (indicated by black arrows) at different time intervals (from 1 d up to 14 d) following 10 nM rapamycin. The graph (**B**) reports the number of mitochondria, which steadily increased at each time interval following rapamycin administration. Graph (**C**) measures the mitochondrial area, which steadily decreased following rapamycin. This indicates that rapamycin produces an augmentation in the number of mitochondria, which feature a much smaller size. Values are the mean ±S.E.M. from 50 cells per group, while mitochondrial area was calculated from 100 mitochondria per group. * *p* ≤ 0.05 vs. Control. Scale bar = 0.56 μm.

Rapamycin at 10 nM increases by two-fold MTR-G immune-fluorescence, which is consistent across U87MG (Figure 6A,B) and A172 (Figure S1A,B) cell lines. It is remarkable that at each time interval, a similar, steady increase in the MTR-G signal was documented for both U87MG (Figure 6) and A172 cell lines (Figure S1). These effects match the data obtained with TEM (Figure 3, Figure 4, Figure 7 and Figure S2). Once the plateau at 24 h was reached, the number of mitochondria persisted unmodified at longer time intervals (up to 14 d) following rapamycin withdrawal without any noticeable decay in either cell line (Figure 7, Figure S2).

### 2.3. Rapamycin Modifies Mitochondrial Number, Size, and Phenotype 

In line with pilot studies reported in Figure 1, Figure 2, Figure 3 and Figure 4, rapamycin at the dose of 10 nM increases remarkably (roughly two-fold of Control) the number of mitochondria in both cell lines (Figure 7 for U87MG and Figure S2 for A172). Such an effect persists following rapamycin withdrawal from 1 d up to 14 d (Figure 7B for U87MG, and Figure S2B for A172). From the representative pictures, the occurrence is evident of well-conformed, densely packed, small mitochondria (Figure 7A for U87MG and Figure S2A for A172), which is consistent with the original descriptions of shape and density of these young organelles [36,37,43,47,48]. The small size of these abundant mitochondria, as directly established by measuring mitochondrial number (Figure 7B and Figure S2B) and mitochondrial area (Figure 7C and Figure S2C), leaves the total area of the cytosol filled with mitochondria of rapamycin- treated cells similar to Controls. In fact, despite the mitochondrial number in rapamycin treated cells being double the Control, the mean mitochondrial area was decreased roughly two-fold compared with Controls. 

**Figure 8:**
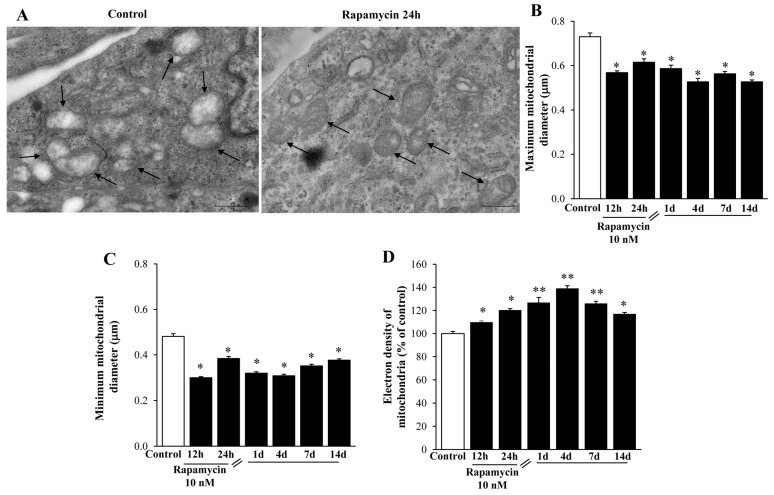
Rapamycin re-shapes mitochondria within U87MG cells at different time intervals. (**A**) Representative TEM micrographs showing mitochondria (arrows) from Control and following 10 nM rapamycin at 24 h. Graphs (**B**,**C**,**D**) confirm that reported in Figure 7 after rapamycin withdrawal from 1 d up 14 d. In fact, both the maximum (**B**) and minimum (**C**) mitochondrial diameter were decreased, whereas the mitochondrial electron density (**D**), which is expressed as a percentage of mitochondrial electron-density measured from Control, was increased. Values were obtained from 100 or 250 mitochondria per group. * *p* ≤ 0.05 vs. Control; ** *p* ≤ 0 05 vs. Control and 12 h. Scale bar = 0.56 μm.

These mitochondria are further shown in Figure 8 and Figure S3. In detail, representative micrographs of Figure 8A and Figure S3A show that rapamycin increases mitochondrial electron density. This is compatible with both maximum (Figure 8B and Figure S3B) and minimum (Figure 8C and Figure S3C) mitochondrial diameter. In fact, both diameters are reduced and persist shorter than Controls for a long time interval following rapamycin withdrawal. In line with this, the electron density of small mitochondria is markedly and persistently increased by rapamycin (Figure 8D and Figure S3D). This suggests that rapamycin promotes the synthesis of young organelles, which indeed, are known to be small in size and surface while possessing an electron- dense matrix and densely packed cristae. These structural features were originally described by the gold-standard reference textbook by Ghadially [47] and they were confirmed by Natale et al. [37], who showed in situ, within neurons the occurrence of mitochondriogenesis and mitochondrial DNA synthesis with TEM following autophagy activation. Thus, rapamycin induces a steady and long-lasting increase in these small, well-shaped mitochondria, which persists at least for 14 d of withdrawal. This suggests mitochondrial long-term plasticity, which is triggered simply by a few hours of mTOR inhibition.

### 2.4. Rapamycin Increases the Expression of Mitochondriogenesis-Related Genes and Constitutive Mitochondrial Genes

**Figure 9:**
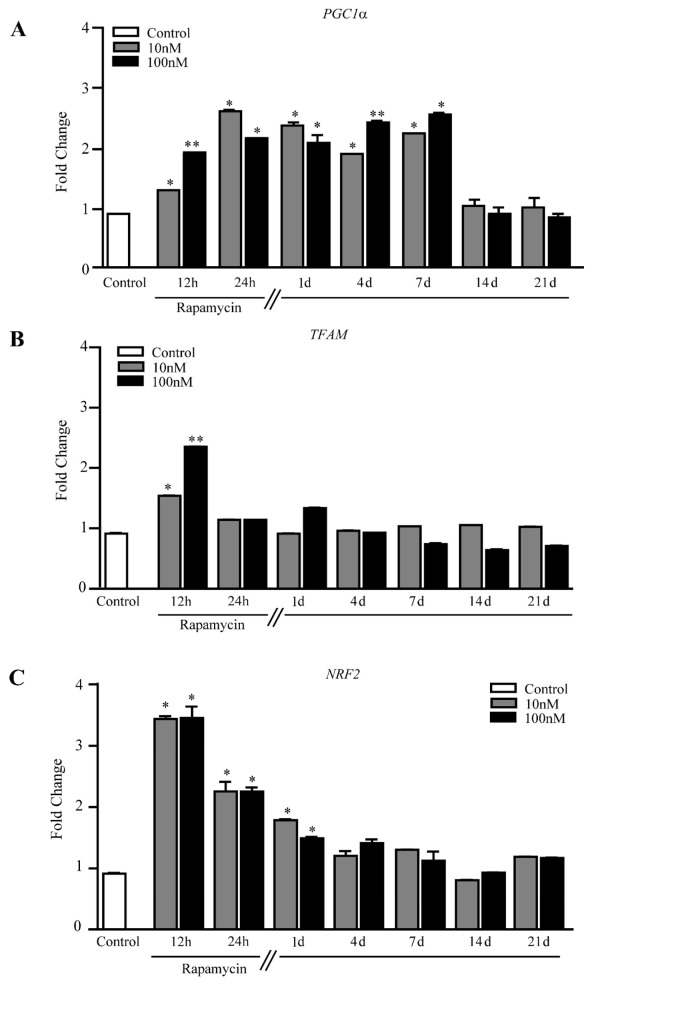
Rapamycin increases mitochondriogenesis-related genes within U87MG cells. RT-PCR for (**A**) *PGC1α*, (**B**) *TFAM*, and (**C**) *NRF2*. * *p* ≤ 0.05 vs. Control; ** *p* ≤ 0.05 vs. Control and 10 nM rapamycin. Each RT-PCR was performed in triplicate and confirmed in two independent experiments using both beta-globin and beta-actin as internal references.

Rapamycin increases *PGC1α*, which induces mitochondrial biogenesis [49] (Figure 9A and Figure S4A). Such an increase is steady up to 7 d of rapamycin withdrawal, which is in line with the long-lasting appearance of newly formed mitochondria described above. 

This contrasts with the transient increase of *TFAM*, present at 12 h but disappearing at 24 h (Figure 9B), suggesting that such a gene works as a trigger for mitochondriogenesis, rather than sustaining a long-lasting effect.

Since mitochondriogenesis is eventually triggered by autophagy of the mitochondria (mitophagy) [50], we measured *NRF2*, the mammalian analogue of the *Caenorhabditis elegans SKN-1*, which represents a molecular bridge between these effects. *NRF2* increases for 4 d following rapamycin withdrawal, peaking at 12 h during rapamycin (Figure 9C). This effect was slightly different in A172 cells (Figure S4B). This is consistent with the fact that in baseline conditions NRF2 protein is transient and undergoes quick degradation while it persists during stressful conditions, when a steady binding to DNA takes place [51,52,53,54].

**Figure 10:**
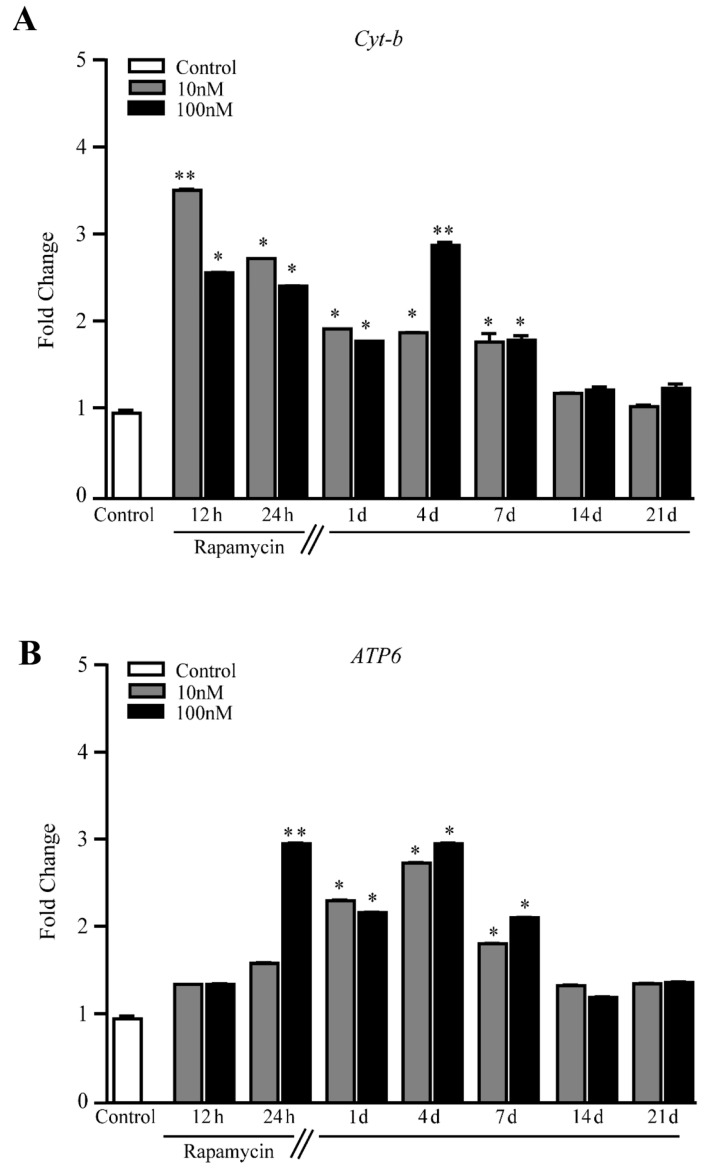
Rapamycin increases mRNA levels for constitutive mitochondrial genes within U87MG cells. RT PCR for (**A**) *Cyt-b,* and (**B**) *ATP6*. * *p* ≤ 0.05 vs. Control. The effects of rapamycin persist up to 7 d of rapamycin withdrawal. ** *p* ≤ 0.05 vs. Control and 10 nM rapamycin. Each RT-PCR was performed in triplicate and confirmed in two independent experiments using both beta-globin and beta-actin as internal references.

When constitutive mitochondrial genes were measured, *Cyt-b* and *ATP6* persistently increased for 7 d following rapamycin withdrawal in U87MG cells (Figure 10A and Figure 10B, respectively) and A172 cells (Figure S5A and Figure S5B, respectively). Thus, as expected, changes in the expression of constitutive mitochondrial genes reproduce the increase in the expression of the gold- standard mitochondriogenesis gene *PGC1α*. These results are consistent with the long-lasting phenotypic changes described above, indicating that the increase in newly synthesized mitochondria is concomitant with the increased gene expression of mitochondrial enzymes. 

**Figure 11:**
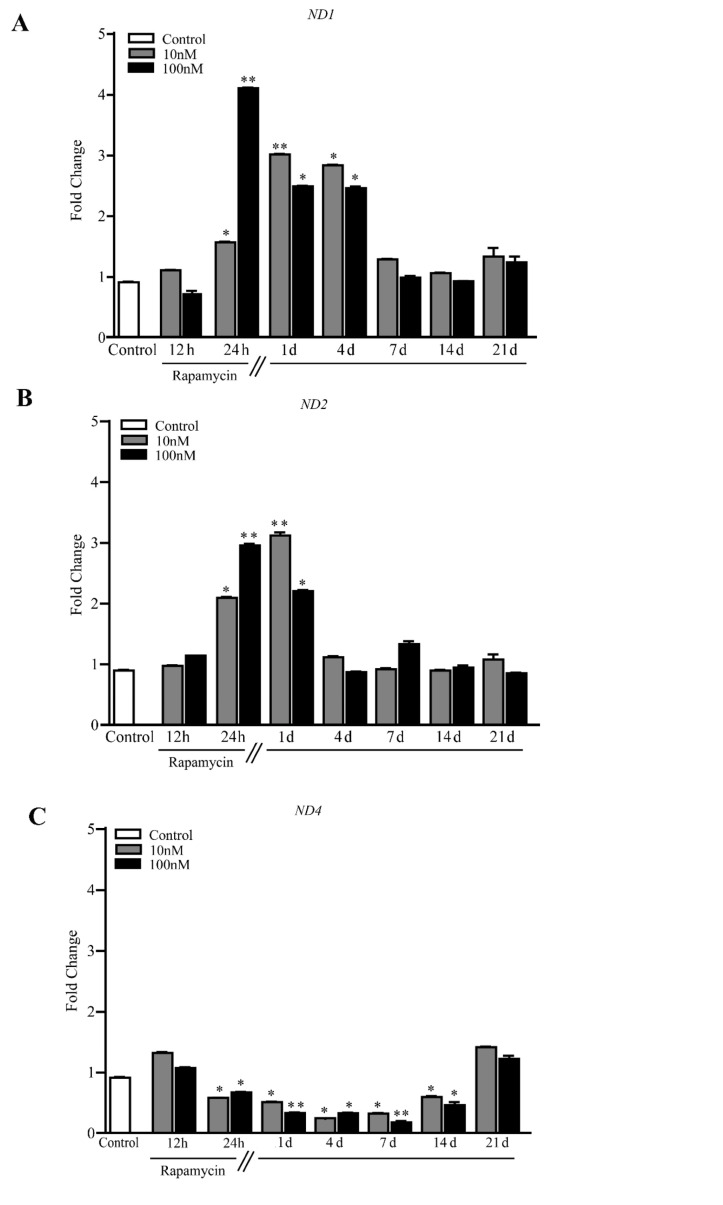
Rapamycin increases mRNA levels for *ND1* and *ND2* while decreasing *ND4* within U87MG cells. RT-PCR for (**A**) *ND1*, (**B**) *ND2* and (**C**) *ND4*. * *p* ≤ 0.05 vs. Control. ** *p* ≤ 0.05 vs. Control and 10 nM rapamycin. Each RT-PCR was performed in triplicate and confirmed in two independent experiments using both beta-globin and beta-actin as internal references.

These findings, in conjunction with the elegant studies by the group of Tavernarakis [50,55,56,57,58] address the crucial issue that autophagy inducers, albeit owning different mechanisms of action such as lithium and rapamycin, similarly increase newly synthesized mitochondria [36,37]. The findings by the group of Tavernarakis [50,55,56,57,58] pose molecular mechanisms linking autophagy to mitochondriogenesis in *C. elegans (SKN-1)*. In the present study, such an interaction was consistent with the quantitative measurement of the mammalian homologue of *SKN-1* which is *NRF2*. The functional significance of producing novel mitochondria is provided by the increase in constitutive mitochondrial genes, such as *Cyt-b, ATP6* and also *ND1* and *ND2* in U87MG cell lines (Figure 11A, and 11B, respectively). Altogether, these constitutive mitochondrial proteins indicate a correct assembly and concrete relevance of mitochondrial biogenesis. 

In detail, *ND1* significantly increases for 4 d following rapamycin withdrawal (Figure 11A), while *ND2* increases for a shorter time interval (Figure 11B). In contrast, *ND4* levels decrease for 14 d following rapamycin withdrawal (Figure 11C). The latter effect is analyzed in the Discussion Section.

**Figure 12:**
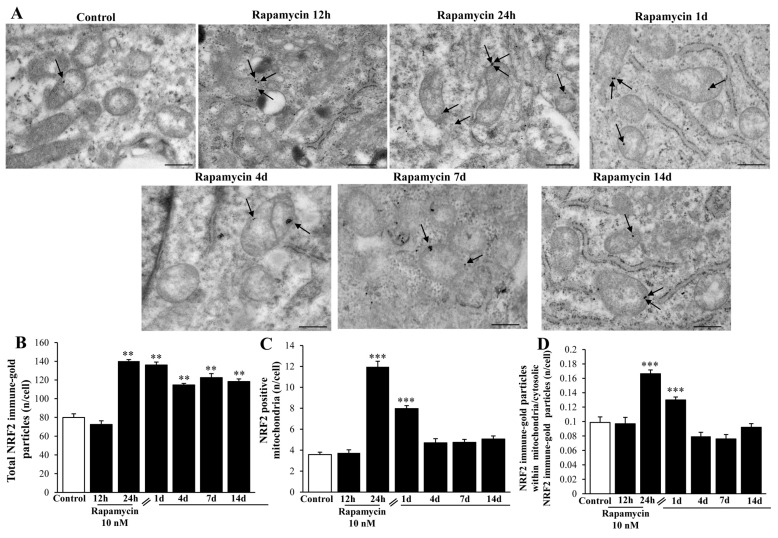
Rapamycin increases the amount of NRF2 immune-gold within U87MG cells. (**A**) Representative TEM micrographs showing NRF2 immune-gold particles (indicated by black arrows) within mitochondria from U87MG cells at different time intervals following rapamycin withdrawal. Graphs report the amount of NRF2 particles per cell (**B**), NRF2-positive mitochondria (**C**), and the ratio of mitochondrial/cytosolic NRF2 particles (**D**). Values were obtained from 50 cells per group. ** *p* ≤ 0.05 vs. Control and 12 h, *** *p* ≤ 0.05 vs. other groups. Scale bar = 0.8 μm.

The levels of NRF2 protein within the cytosol and mitochondria were measured through immune-gold staining with TEM. The total amount of NRF2 per cell was markedly increased up to 14 d following rapamycin withdrawal, although at 12 h during rapamycin (when *NRF2* mRNA levels were already elevated) the protein levels were similar to Control (Figure 12A,B). The specific placement of NRF2 at mitochondrial level was dramatically increased at 24 h of rapamycin and 1 d following rapamycin withdrawal (Figure 12C). When the ratio of total vs. mitochondrial NRF2 was calculated, the polarization of the protein towards mitochondria was still significant at 24 h and 1 d (Figure 12D). Such a discrepancy for the whole cell increase in NRF2 (long-lasting) and the site-specific increase within mitochondria (transient, at 24 h of rapamycin and 1 d following rapamycin) is presently unknown, although it suggests a more generalized effect on organelle biogenesis going beyond mitochondrial level. This was confirmed also in the A172 cell line (Figure S6).

**Figure 13:**
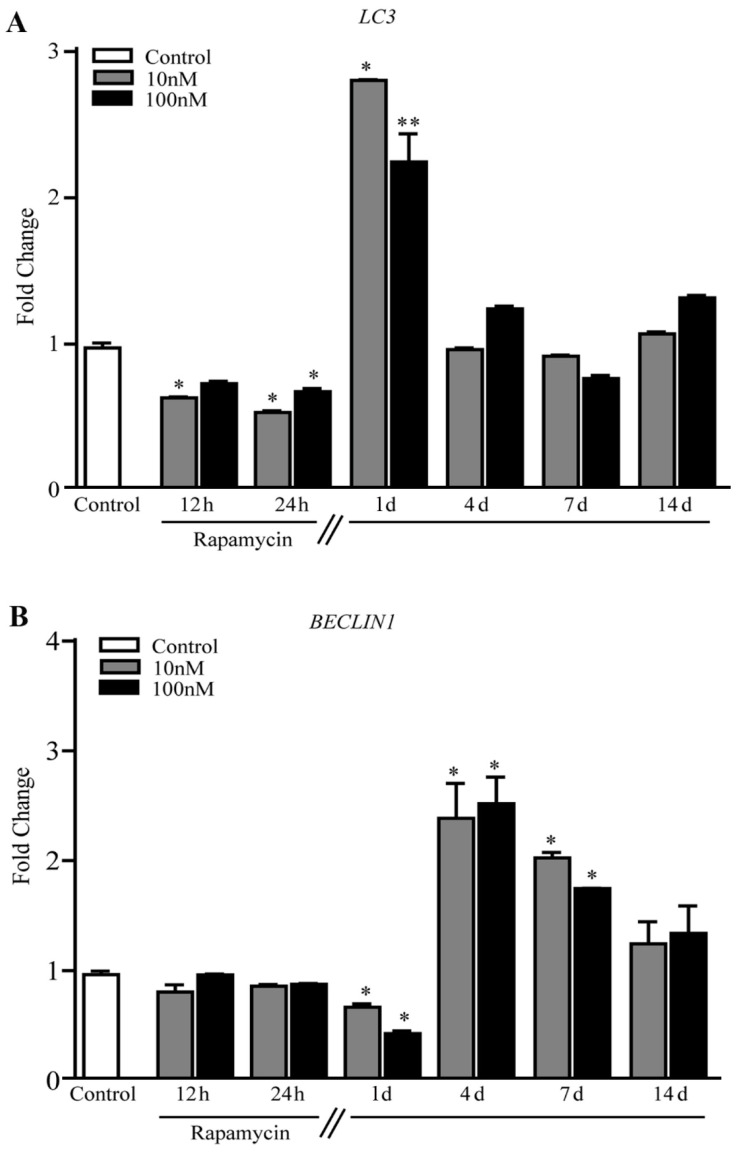
Rapamycin increases the expression of autophagy related genes in U87MG cells. (**A**) RT-PCR for *LC3*, and (**B**) RT-PCR for *BECLIN1.* * *p* ≤ 0.05 vs. Control. ** *p* ≤ 0.05 vs. Control and 10 nM rapamycin. Each RT-PCR was performed in triplicate and confirmed in two independent experiments using both beta-globin and beta-actin as internal references.

The present findings indicate the occurrence of mitochondriogenesis, which needs to be analyzed in association with the activation of autophagy (and mitophagy) within mTOR-overexpressing GBM cells. In fact, the autophagy-related gene *LC3* increases by three-fold that of Control at 1 d following rapamycin (Figure 13A). Not surprisingly, the levels of *BECLIN1* under the effects of rapamycin followed a similar trend (a decrease followed by an increase) with a shift towards longer time intervals, being still elevated at 7 d (Figure 13B). 

### 2.5. Immune-Fluorescence of LC3 within U87MG and A172 Cells

**Figure 14:**
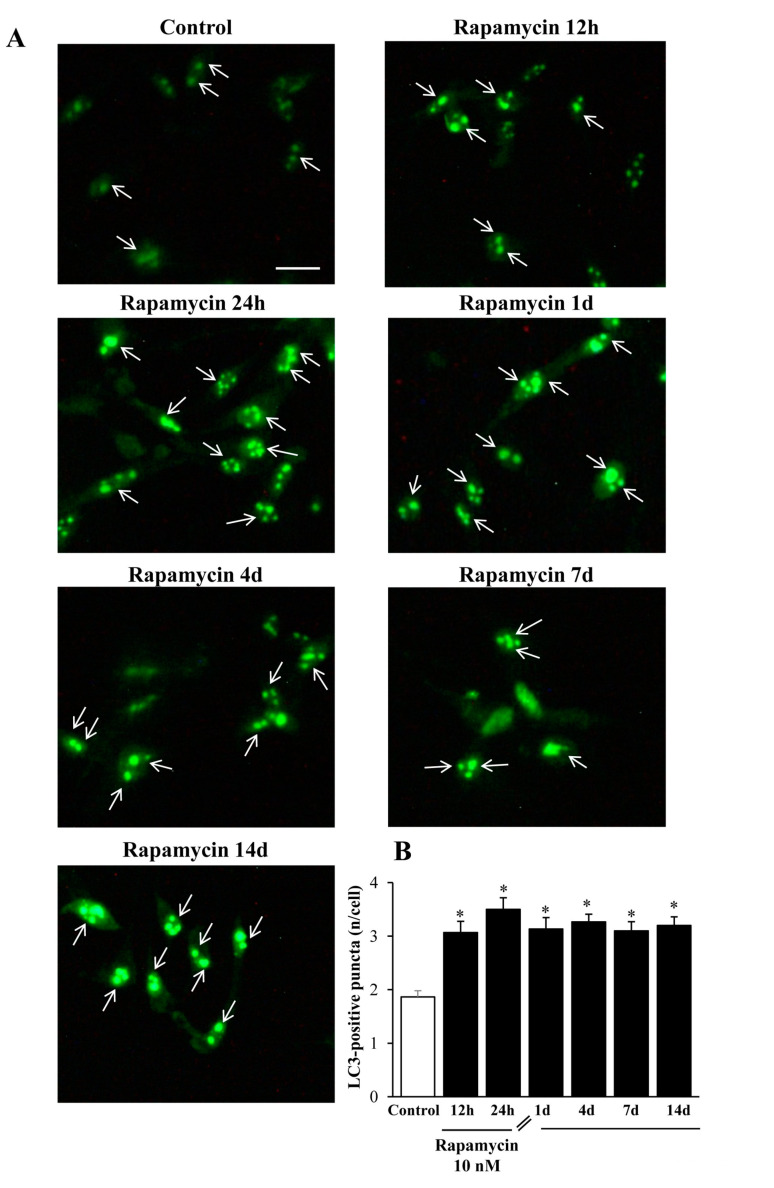
Rapamycin increases the immunofluorescence of LC3 within U87MG cells. (**A**) Representative immunofluorescence from U87MG cells. White arrows point to LC3 puncta (green). (**B**) Graph reports the number of LC3 puncta per cell. Values were obtained from 100 cells per group. * *p* ≤ 0.05 vs. Control. Scale bar: 20 μm.

When rapamycin was administered for 24 h, the classic autophagy marker LC3 protein suddenly increased in line with its gene expression trend (representative pictures of Figure 14A). Such an increase persisted unmodified for 14 d without any noticeable decay, which surpasses the endurance of that described for the long-lasting increase in the gene expression levels (graph at Figure 14B).

**Figure 15:**
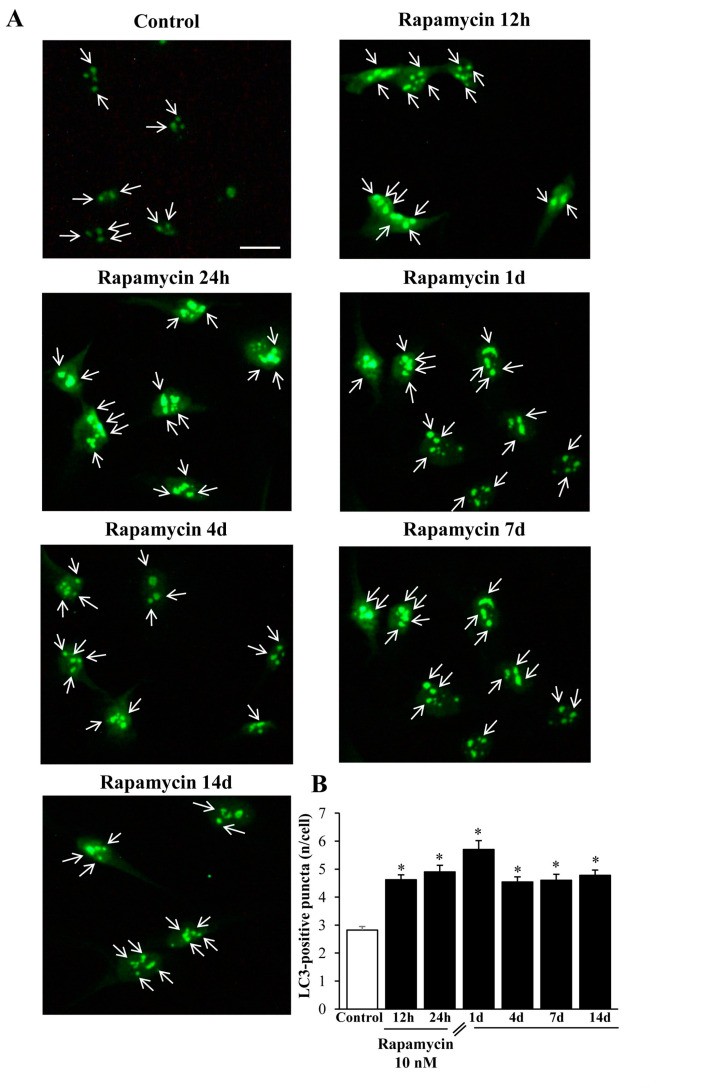
Rapamycin increases the immunofluorescence of LC3 within A172 cells. (**A**) Representative immunofluorescence from A172 cells. While arrows point to LC3 puncta (green). (**B**) Graph reports the number of LC3 puncta per cell. Values were obtained from 100 cells per group. * *p* ≤ 0.05 vs. Control. Scale bar: 25 μm.

Exactly the same effects could be described within A172 cell lines (Figure 15A,B). The authors are routinely reluctant to carry out quantitative measurements based on immunofluorescence (IF) data, mostly due to the non-linear correlation between the antigen amount and the fluorescence signal. Nonetheless, in this case we could observe a clear-cut presence of IF puncta which enabled a fairly confident count of puncta within cell cultures. For such a reason, we counted the amount of LC3-IF puncta in each cell and the counts refer to the mean amount of puncta within single cells to rule out the bias due to variations in cell density promoted by rapamycin.

**Figure 16:**
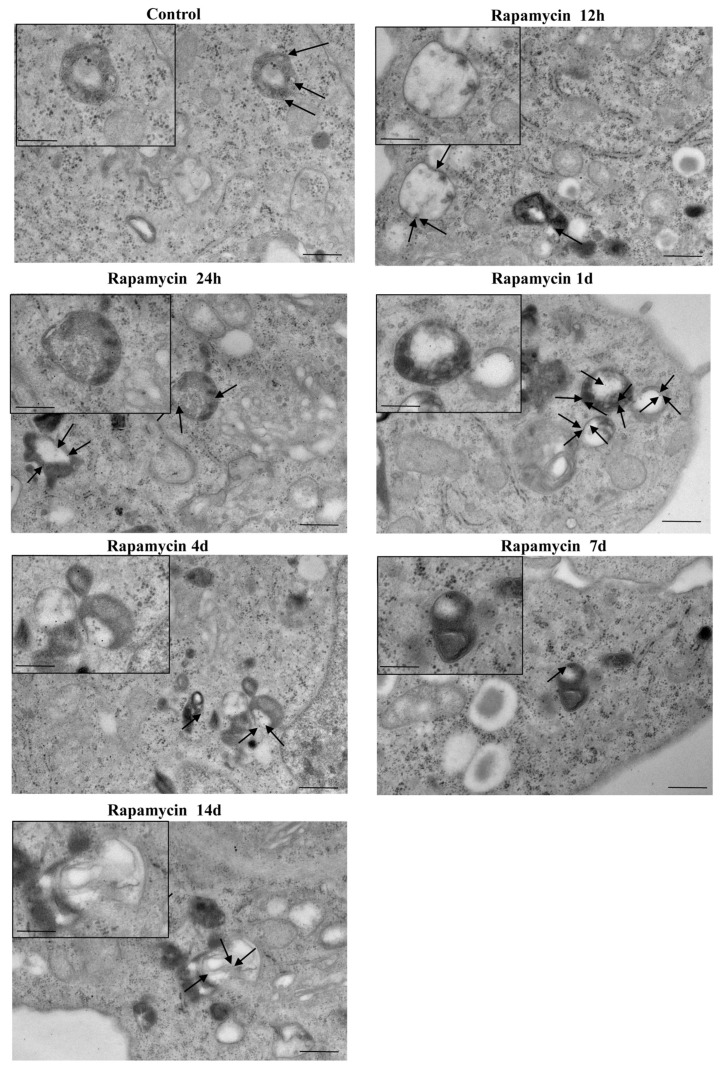
Representative pictures of rapamycin-induced LC3-stained and non-stained vacuoles within U87MG cells. The black box indicates high magnification inserts showing LC3-stained vacuoles. Black arrows point to LC3 immune-gold particles on vacuoles. Micrographs were obtained from 1 cell per group, for a total of 7 cells. Scale bar: 0.6 μm (low magnification); 0.20 μm (high magnification).

**Figure 17:**
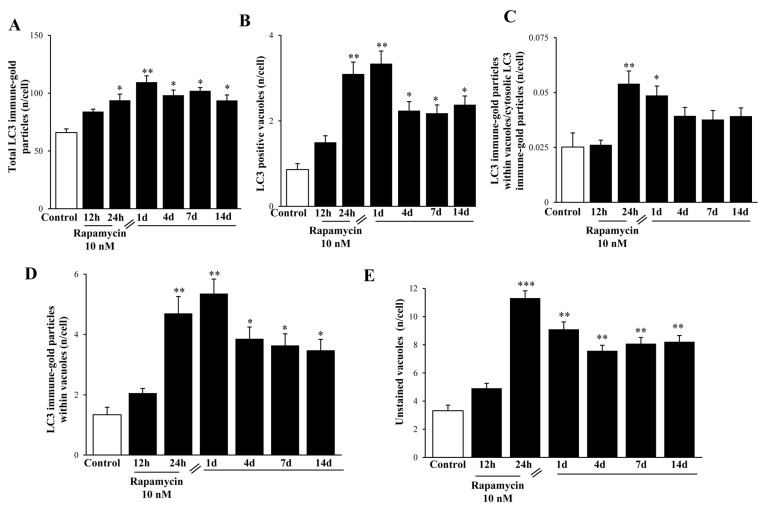
Rapamycin increases the amount of both LC3-stained and non-stained vacuoles within U87MG cells. LC3 immune-gold particles were increased by rapamycin within the cytosol (**A**) and vacuole compartments (**B**). The ratio of vacuolar/cytosolic LC3 was also increased by rapamycin (**C**), which also filled each LC3-positive vacuole with a higher stoichiometric density of LC3 proteins (**D**). Rapamycin increases the vacuolar compartment also considering those vacuoles which do not stain for LC3 (**E**). Values were obtained from 50 cells per group. * *p* ≤ 0.05 vs. Control; ** *p* ≤ 0.05 vs. Control and 12 h rapamycin; *** *p* ≤ 0.05 vs. all other groups.

An internal validation of these immunofluorescent counts is provided by the following figures which deal with the amount of TEM-identified LC3 vacuoles (Figure 16, Figure S7, Figure 17, Figure S8).

### 2.6. In Situ Immune-Gold-Based Assessment of Autophagy 

Stoichiometric measurement of LC3 immune-gold proteins suddenly increases and persists for a long time following rapamycin. This effect is shown at high magnification in the representative Figure 16 (U87MG) and Figure S7 (A172) in the whole cell. The time-course was prolonged, being still detectable at 14 d (Figure 16, U87MG; Figure S7, A172). Such an increase is dramatic when measured either within cytosol (Figure 17A and Figure S8A) or within autophagy vacuoles (Figure 17B,C and Figure S8B,C, respectively). Polarization of LC3 towards autophagy vacuoles was dramatically increased by rapamycin. In fact, in situ imaging allows the detection of a strong vacuolar polarization compared with cytosol, which was maximally expressed at 1 d (Figure 17C and Figure S8C). Moreover, when counting the stoichiometric amount of LC3 protein within LC3-positive vacuoles, this was further increased by rapamycin, thus producing an increase in the density of LC3 protein within increased LC3 immune-stained vacuoles (Figure 17D and Figure S8D). Consistently, the same time interval, 1 d, corresponds to the time when a significant increase of *LC3* gene expression is measured (Figure 13A). This suggests that the great amount of newly synthesized LC3, upon activating stimuli, selectively moves towards specific vacuolar compartments. Thus, rapamycin produces at first a widespread increase of LC3, which is further recruited within autophagy vacuoles. The effects of rapamycin on the autophagy machinery cannot be fully deciphered by counting LC3- positive vacuoles, since at the same time, several vacuoles unstained for LC3 increased dramatically following rapamycin administration (Figure 17E and Figure S8E). The nature of these vacuoles is fascinating and remains to be investigated.

### 2.7. Mitophagy Copes with Autophagy Activation

**Figure 18:**
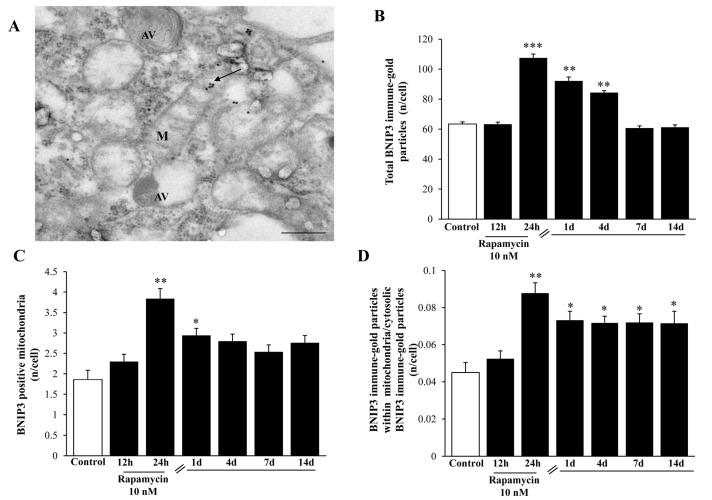
Rapamycin increases the mitophagy marker BNIP3 at short-time intervals within U87MG cells. (**A**) Representative BNIP3-stained mitochondrion (indicated by the black arrow) at 24 h during 10 nM rapamycin, continuous exposure. Graphs report: (**B**) the amount of total BNIP3 particles; (**C**) the number of BNIP3-positive mitochondria; (**D**) the ratio of mitochondrial/cytosolic BNIP3 particles at various time interval during (12–24 h) or following (from 1 d up to 14 d) rapamycin exposure. M = mitochondrion, AV = vacuoles. * *p* ≤ 0.05 vs. Control; ** *p* ≤ 0.05 vs. Control and 12 h; *** *p* ≤ 0 05 vs. other groups. Scale bar = 0.3 μm.

In order to address specifically and quickly the occurrence of mitophagy, we carried out a stoichiometric measurement of BNIP3 immune-gold particles within U87MG cells. In line with measurement of plain autophagy markers, we found that rapamycin increased BNIP3 in the GBM cells, as shown in the representative picture in Figure 18A (which refers to the effects observed at 24 h of rapamycin exposure). The increase in BNIP3 counted in the cytosol persisted from 1 d up to 4 d following rapamycin treatment (Figure 18B). Such an increase was shorter-lasting (only 1d following rapamycin) at mitochondrial level (Figure 18C), which suggests other potential compartments targeted by this protein as confirmed by the calculation of the polarization of BNIP3 within mitochondria where it persisted elevated at least for 14 d (Figure 18D). 

### 2.8. Autophagy Activation Includes Merging with Lysosomes 

**Figure 19:**
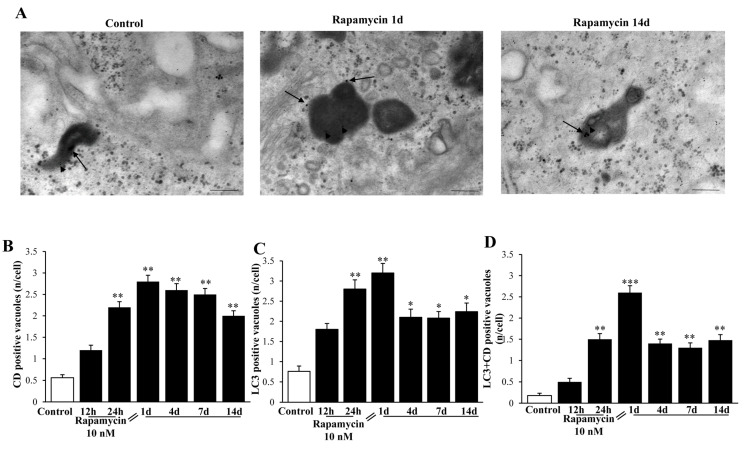
Rapamycin increases LC3+Cathepsin-D co-immune-stained vacuoles within U87MG cells. (**A**) Representative TEM micrographs show LC3+Cathepsin-D co-immune-stained vacuoles within Control cells and following 1 d and 14 d of rapamycin withdrawal. Arrowheads and black arrows point to LC3 (10 nm) and Cathepsin-D (20 nm) immune-gold particles, respectively. Graphs report the time-course of rapamycin-induced increase in (**B**) Cathepsin-D (CD) only immune-gold stained vacuoles; (**C**) LC3 only immune-gold stained vacuoles; (**D**) LC3 + Cathepsin-D immune-gold double-stained vacuoles. Rapamycin increases each vacuole immune-gold staining compared with Control. However, the effects produced on double-stained vacuoles exceeded at large what measured for single staining, which indicates a rapamycin-dependent autophagy progression toward lysosome. * *p* ≤ 0.05 vs. Control; ** *p* ≤ 0.05 vs. Control and 12 h. Scale bar: 0.2 μm.

Despite technical issues, which need to be considered concerning the staining with Cathepsin-D immune-gold particles in a context of high background electron-density within lysosomes, we were able to measure a rapamycin-induced increase in Cathepsin-D-stained electron-dense vacuoles (representative Figure 19A). In detail, we carried out a quantitative morphometry for single Cathepsin-D immune-stained vacuoles shown in the graph in Figure 19B. Single LC3 immune- stained vacuoles are reported in the graph in Figure 19C. Rapamycin induces an increase in LC3 vacuoles approaching that previously reported (roughly 400% of Control as maximal increase, at 1 d of rapamycin withdrawal). Such an increase was similar for Cathepsin-D immune-stained vacuoles (roughly 500% of Control as maximal increase at 1 d of rapamycin withdrawal). Nonetheless, when co-immune-stained vacuoles were counted, rapamycin produced a 1400% increase of Controls at 1 d of rapamycin withdrawal (Figure 19D). This indicates that despite increasing both LC3 vacuoles and Cathepsin-D immune-stained vacuoles, the effects of rapamycin compared with Control are enhanced when measuring LC3 + Cathepsin-D co-immune-stained vacuoles. Such an increase largely exceeded what was expected by the increase produced for each antigen alone (either LC3 or Cathepsin-D). This suggests a specific effect of rapamycin in GBM cells in promoting a long-lasting progression of autophagosomes to merge with lysosomes. 

### 2.9. The Role of mTORC1 in the Effects Induced by Rapamycin in GBM Cell Lines

A number of additional studies are required to provide final proof of the specific role of mTORC1 in all these effects induced by rapamycin in GBM cells. These include a number of genetic manipulations, which require several experiments to be carried out. The most direct, though semi- quantitative approach to check the efficacy of mTORC1 inhibition under the effects of rapamycin in the present experimental conditions was the measurement of mTORC1 downstream products. In fact, we carried out immune-blotting of phopspho-p85 S6K and phopspho-p70 S6K following the same time-course of 10 nM rapamycin administration used for all experimental data provided so far. Immune-blotting analysis indicated that rapamycin (10 nM) produces a long-lasting inhibition of mTORC1 activity in GBM cells (Figure S9). In future experiments we are planning to replicate all these data in vitro and in vivo to dissect each step operating in each molecular effect shown here through specific genetic manipulation. This can be achieved by overexpressing or silencing genes of mitochondriogenesis, or genes required to synthesize the mTORC1 complex as well as specific, step-dependent autophagy and mitophagy genes. While measuring the efficacy of gene silencing in these experimental conditions, other studies on genetic manipulation of lysosomal activity are needed. 

## 3. Discussion and Conclusions

The present research study quantifies various facets of autophagy-associated mitochondriogenesis in mTOR up-regulated GBM cells. The manuscript focuses on the effects produced following mTOR-inhibition and subsequent autophagy stimulation induced by rapamycin. In fact, GBM cells in baseline conditions feature marked autophagy suppression, which is due, at least in part, to mTOR up-regulation. In the literature, evidence is provided showing that GBM is characterized by autophagy dysfunction which can be rescued by mTOR inhibition. It is postulated that when autophagy is activated, mitochondriogenesis is synergistically stimulated [57,58]. However, as remarked recently by Palikaras et al. [57] there is a strong need to understand the way in which autophagy and mitochondrial biogenesis coordinate each other [57,58]. 

The present research paper follows such a line, which fulfils the need to dissect those events, which tune the balance between autophagy and mitochondrial biogenesis along with the correct assembly of mitochondrial DNA [58]. In the present study the occurrence of autophagy and mitochondrial biogenesis were quantified in mTOR up-regulated cells and under the pharmacological effects of mTOR inhibition. Quantitative evidence was provided by LC3 immunofluorescence, mitotracker-green, and expression of autophagy- and mitochondriogenesis- related proteins and genes. The coordination of these processes under pharmacological manipulation was evidenced, as well as the progression towards the Cathepsin-D immune-stained lysosomal compartment. The occurrence of mitochondrial biogenesis is consistent with the increase in the mitochondrial number as well as the reduction in mitochondrial area. This correlates with a reduced maximum and minimum mitochondrial diameter. These phenotypic changes, along with a greater electron-density and densely packed cristae are typical of newly generated organelles [37,47]. They correspond to the mitochondria shown here under the long-lasting effects of rapamycin, which possess indeed an increased electron-density, mostly due to densely packed cristae and a rich, well-assembled matrix content. The present study strongly correlates this phenotype to concomitant molecular events at gene and protein level. In fact, mitochondrial remodelling correlates with increased expression of those genes which stimulates mitochondriogenesis. These genes lead to the correct assembly of constitutive mitochondrial DNA, consistently with the remarkable shape developed by these organelles. This is concomitant with an increase in mitochondriogenetic proteins counted in situ, stoichiometrically, within newly formed mitochondria. Altogether, these findings indicate that rapamycin promotes effective mitochondrial biogenesis evidenced by an increased number and magnificent structure. When describing the cell pathology affecting GBM, an autophagy defect and a mitochondrial dysfunction are reported as independent phenomena. On the other hand, as shown here, both phenomena are grounded on similar molecular events. In fact, when rapamycin was administered, a dramatic activation of autophagy machinery takes place, as measured by the amount of both *LC3* and *BECLIN1* as well as the number of unstained vacuoles. Remarkably, LC3 particles, which were increased in the whole cytosol by rapamycin administration, suddenly polarize to vacuoles. These molecular events associate with an improvement of mitochondrial phenotype reported above, which is consistent with the up-regulation of specific genes such as *PGC1α*, *TFAM*, and *NRF2*. These data confirm in mammalian GBM cells that found by Palikaras et al. [50] in *C. elegans* about SKN-1 protein, which links mitophagy to the biogenesis of mitochondria. In fact, in the present study, the SKN-1 mammalian homolog NRF2 was increased by rapamycin both upstream at genetic level and downstream as protein in situ within mitochondria. NRF2 shuttles the signal for mitochondriogenesis from mitophagy-prone altered mitochondria towards the nucleus to induce mitochondrial biogenesis [50,56]. This undoubtedly confirms in human GBM cells that mitochondrial biogenesis and mitophagy need to be coupled through a molecular interplay in order to preserve homeostasis as postulated by Palikaras and Tavernarakis [55]. Here, in mammalian GBM cells under the effects of rapamycin, autophagy was concomitant with the genetic stimulation of mitochondriogenesis. It looks as if the need to amplify a defective compartment (failure in mitochondrial activity) is suddenly faced by the cell, which tries to compensate by increasing mitochondrial number. Such an attempt owns inherent limits due to the persistence of defective mitochondrial matter. The biogenesis of novel mitochondria circumvents this defect. To measure the potential efficacy of such a process we detected constitutive mitochondrial enzymes such as *Cyt-b*, *ATP6*, *ND1,* and *ND2*, which witness a functionally operative mitochondrial biogenesis. 

Although constitutive mitochondrial genes *ND1* and *ND2* were increased following rapamycin administration, the effects of rapamycin on *ND4* levels were confused. In fact, apart from a slight increase at 12 h, a persistent significant decrease in *ND4* levels was measured up to 14 d. The reason for such a discrepancy was not analyzed in the present experimental work. It may be hypothesized that, since *ND4* is placed in a locus where mtDNA is more vulnerable to structural re-arrangements [59,60], this may lead to a loss of integrity upon mtDNA replication (Figure S10).

It is remarkable that a number of molecules which were analyzed in this study as part of specific pathways involved in mitochondrial dynamics, were modified persistently and harmonically following rapamycin administration. This is even more significant when considering the up- regulation of specific genes which remain elevated even for at least 14 d following rapamycin withdrawal. The persistency of rapamycin-induced gene expression translates into persistent protein and organelle changes, which provides strong evidence for autophagy-related plasticity. This is reminiscent of long-lasting plastic effects on mitochondrial morphology persisting for months following other autophagy inducers such as lithium [36,37,48]. Since lithium activates autophagy independently of mTOR, this suggests that autophagy per se (induced whatsoever) produces plastic changes in mitochondrial biogenesis, fine morphology, and constitutive assembly, which persist way beyond the presence of whatsoever is triggering stimulus. 

In summary, these findings provide a further piece of evidence applied specifically to the pathogenic pathways in human GBM cells, which is consistent with that hypothesized more than a decade ago [36,37,61]. In fact, as mitochondria are highly dynamic organelles owning a function which is crucial for cell homeostasis, stemness, and tumorigenesis, the tight regulation of those pathways that regulate mitochondrial content and metabolism appears mandatory for cell survival, growth, and differentiation. 

Despite being replicated in two different cell lines of GBM and despite being exciting, the significance of the present findings should be carefully considered and honestly toned down accordingly, as they were obtained in the context of cell models. 

## 4. Materials and Methods 

### 4.1. Experimental Design

Experiments were carried out on U87MG and on A172 human glioblastoma cells. 

The U87MG cell line was obtained from Cell Bank (IRCC San Martino-IST, Genova, Italy). The cells were maintained in DMEM growth medium (Sigma-Aldrich, Saint Louis, MO, USA) containing 10% Fetal Bovine Serum (FBS, Sigma-Aldrich), 1% of MEM Non-Essential Amino-Acid (MEM-NEAA, Sigma-Aldrich), penicillin and streptomycin (50 IU/mL and 100 μg respectively, Sigma-Aldrich) and kept at 37 °C in a humidified atmosphere containing 5% CO_2_. 

The A172 cell line was obtained from the European Collection of Authenticated Cell Cultures (ECACC) and from Cell Bank (IRCC San Martino-IST, Genova, Italy) and maintained in Modified Eagle’s Medium (Euroclone, Milan, Italy) supplemented with 10% FBS, 2 mM L-glutamine, 100 IU/mL penicillin and 100 mg streptomycin, at 37 °C, 5% of CO_2,_ and 95% of humidity.

We carried out pilot experiments by using three doses of rapamycin (1, 10, and 100 nM). To select the dose needed to produce mitochondrial effects we roughly focused on a few measurements (changes in the number of mitochondria and changes in mitochondrial size). These measurements are key in the light of the experimental aim, which consists of measuring the mitochondriogenesis associated with mTOR-dependent autophagy. We chose to expose cells to rapamycin for short-time intervals to assess early effects and to establish whether long-term, autophagy-dependent mitochondrial biogenesis lasts for weeks after rapamycin withdrawal. Since multiple methods were used to assess mitochondrogenesis, one may argue that the optimal dosing of rapamycin may differ depending on which specific approach was applied. Remarkably, we documented in these pilot trials that a dose of 10 nM rapamycin was fully effective to detect mitochondriogenesis independently of the specific experimental procedure. This was preliminarily established by combining the counts of mitochondrial number (Figure 1, Figure 2, Figure 3 and Figure 4). These preliminary investigations were needed to proceed with the actual experimental phase, which measured specifically whether rapamycin exposure induces mitochondriogenesis through gene and site-specific protein expression. Details on the preliminary experimental concepts were obtained concerning mitochondrial number. When an increased number of small and well-conformed mitochondria was documented, it was likely that mitochondrial biogenesis was taking place [37]. Nonetheless, this inference remained rather hypothetical in the absence of specific markers witnessing for mitochondrial biogenesis. Therefore, we marked specific pathways to assess the occurrence of biogenesis under autophagy activation, following the effects of rapamycin compared with control conditions. It is remarkable that independently of the specific techniques, data concerning timing and dosing of rapamycin on mitochondria strongly overlapped, which was an internal validation of the strength of the present findings.

#### Considerations on Pilot Experiments Assessing the Effects of Various Doses and Times of Rapamycin on the Mitochondrial Number

The data from Figure 1 and Figure 2 indicate that rapamycin at 10 nM reaches a steady effect on mitochondrial number. Thus, the effects produced by a dose of rapamycin at 10 nM correspond to a steady effect, which was evident at each time interval being tested and reported in Figure 3 and Figure 4. However, in the time course, such an increase was more pronounced at 24 h of rapamycin compared with 12 h and 72 h as shown in Figure 3B and Figure 4B. Thus, the 24 h time interval was more effective in rising the mitochondrial number (two-fold of Controls). Based on these data we planned the core experimental steps by using a stimulus of 10 nM rapamycin for 24 h to assess a number of issues at various time intervals from 1 d up to 7 d of rapamycin withdrawal.

### 4.2. RNA Extraction 

Total RNA was isolated from cultured cells using TRIzol Reagent (Invitrogen, Life Technologies, Waltham, MA, USA) according to the manufacturer’s instructions. The concentration and purity of RNA samples were determined using Nanodrop 2000 (Thermo Scientific, Life Technologies, Waltham, MA, USA). Total RNA (100 ng) was reverse transcribed (RT) with SuperScript^®^ VILO^TM^ (Invitrogen, Life Technologies, Waltham, MA, USA) according to the manufacturer’s instructions.

### 4.3. qReal-Time-PCR 

Amplifcation and detection were performed on a CFX Connect^TM^ Real Time System (Bio-Rad, Hercules, CA, USA). PCR mix including 10 μL SYBR Green PCR Master (Applied Biosystems, Foster City, CA, USA), 0.5 μM of each primer and 0.8 μL of RT reaction mix, was amplified as follows: 50 °C for 1 min, 95 °C for 10 min followed by 40 cycles of 95 °C for 30 s, 54 °C for 1 min. The primers (Table 1) were designed using GenBank (http://www.ncbi.nlm.nih.gov/). 

Positive controls (DNA), negative control (distilled water), and RT-negative controls (total RNA sample) were included in each run. 

The relative quantification was calculated using the comparative Ct method (also known as the ΔΔCT method) [62,63], and beta-globin and beta-actin were selected as internal references. Ct values correspond to mean values of each PCR performed in triplicate. Gene expression was confirmed in two independent experiments using both beta-globin and beta-actin as internal references. Real time PCR was carried out for the following genes to measure different steps of mitochondrial dynamics:

Mitochondrial biogenesis was related to specific genes (*PGC1α; TFAM; NRF2*) as well as constitutive mitochondrial genes (*ND1; ND2; ND4; Cyt-b; ATP6*). After a short challenge, the experimental design proceeded in the absence of rapamycin at 1 d, 4 d, 7 d, 14 d, and 21 d (only for gene expression, otherwise the longest time of withdrawal was 14 d) of rapamycin withdrawal. The actual experimental phase was based on the use of specific markers to measure mitochondriogenesis with specific markers reported above and a detailed morphometric quantification of mitochondrial number size and structure along with the dynamics of autophagy vacuoles, through the gold-standard (BECLIN1/LC3 stained double membrane vesicles having electron-density similar to cytosol). 

To assess the short-term effects produced by mTORC1 modulation, cells treated with rapamycin (10 nM or even 100 nM, the highest dose only for gene expression) were harvested at 12 h or 24 h.

To assess the long-lasting effects (at 1 d, 4 d, 7 d, 14 d, up to 21 d following a single 24 h shot of rapamycin 10 nM) the culture medium was replaced at first 24 h after the shot of rapamycin and subsequently every three days with fresh medium. Dilutions of rapamycin were obtained from a stock solution of 1 mM of rapamycin in 1.41 M Dimethyl Sulfoxide (DMSO), which was dissolved in culture medium with a final concentration of 0.01% DMSO. Control cells were maintained in culture medium containing 0.01% DMSO.

### 4.4. Transmission Electron Microscopy (TEM)

Cells (10^6^) were seeded in 10 mm diameter culture dishes with 5 mL of culture medium. After removing culture medium, the first fixing solution (2.0% paraformaldehyde/0.1% glutaraldehyde in 0.1 M PBS pH 7.4) was added to cell culture for 90 min at 4 °C. Cells were gently scraped from the plate and centrifuged at 10,000 rpm for 10 min to obtain the cell pellet. The pellet was washed in PBS, centrifuged and post-fixed in 1% osmium tetroxide (OsO_4_) for 1 h at 4 °C; the pellet was dehydrated using increasing ethanol concentrations and finally embedded in epoxy resin. 

OsO_4_ interacts with lipids preserving membranes from artifacts. Fixing and post-fixing solutions and the use of epoxy resin were validated in our previous studies for immune-gold-based ultrastructural morphometry [37,64,65,66]. In fact, a combination of aldehydes, OsO_4_, and epoxy resin allows a minimal epitope covering, while preserving sub-cellular architecture. In particular, OsO_4_ binds to cell membranes, thus enhancing the contrast of cytosolic compartments while preventing membrane artifacts. Moreover, epoxy resin better preserves cell morphology compared with acrylic resin; again, it enhances stability of the sections in the electron beam, and aids section cutting through the blocks trim.

Ultrathin sections were cut at ultramicrotome (Leica Microsystems, Wetzlar, Germany) and they were dedicated to plain electron microscopy or post-embedding immune-electron microscopy. Ultrathin sections were observed at Jeol JEM SX100 electron-microscope (Jeol, Tokyo, Japan). 

#### 4.4.1. Post-Embedding Immune-Electron Microscopy 

Immune-electron microscopy can be defined as TEM combined with secondary antibody used for immune-localization of antigens in different cell compartments. Post-embedding immune-electron microscopy was carried out to test different antibodies in ultrathin sections cut from the same resin-embedded sample block. Ultrathin sections were collected on nickel grids and processed for protein detection detailed in Table 2, along with the source (RRID) of each antibody. Control sections were obtained by incubating with the secondary antibody only. This procedure is a technical challenge requiring optimization of tissue fixation and embedding methods. The use of gold-conjugated secondary antibodies allows high resolution, detection, and localization of the stoichiometric antigen-antibody reaction in sub-cellular structures. In particular, the gold-conjugated particles allow morphometric analysis and detection of proteins placement in different compartments, such as autophagy vacuoles and mitochondria. For the removal of OsO4, nickel grids were incubated with a saturated NaIO_4_ solution, which is recommended for antigen unmasking, maintaining a good ultrastructural detail [67]. The sodium metaperiodate is an oxidizing agent which attacks the hydrophobic alkane side-chains of epoxy resin making the sections more hydrophilic and allowing a closer contact between immune-gold-conjugated antibodies and the antigens exposed on the surface of each section [37].

Here we report the rough list of primary antibodies used: (i) nuclear respiratory factor 2 (NRF2, Abcam) marker of mitochondrial biogenesis; (ii) microtubule-associated protein 1 A/1B-light chain 3 (LC3, Abcam, Cambridge, UK), the gold standard marker to identify autophagy vacuoles; (iii) Bcl-2/adenovirus E1B 19 kDa interacting protein 3 (BNIP3, ThermoFisher, Waltham, MA, USA) marker of mitophagy and (iv) Cathepsin-D (CD, Sigma-Aldrich) marker of lysosomes.

In detail, grids were incubated on drops of blocking buffer (10% goat serum and 0.2% saponin in PBS) for 20 min at 21 °C, and then, with a single primary antibody (LC3 diluted 1:50, NRF2 diluted 1:20, BNIP3 diluted 1:15 and Cathepsin-D diluted 1:10) or in combination (LC3 diluted 1:50 and Cathepsin-D diluted 1:10). 

Incubations were carried out on drops of ice-cold PBS containing 1% goat serum and 0.2% saponin in a humidified chamber overnight at 4 °C. Then, ultrathin sections were washed in cold PBS, before applying the gold-conjugated secondary antibodies (10 nm gold particles; 20 nm gold particles, BB International, Treviso, Italy), diluted (1:20) in blocking buffer (1% goat serum and 0.2% saponin in PBS) for 1 h at 21 °C. After rinsing in PBS, grids were incubated with 1% glutaraldehyde for 3 min, they were washed in distilled water to remove traces of salts and prevent precipitation of uranyl-acetate, and they were counterstained with a saturated solution in distilled water of uranyl acetate and lead citrate to be finally observed by using a Jeol JEM SX100 electron-microscope (Jeol, Tokyo, Japan).

#### 4.4.2. Ultra-Structural Analysis of Mitochondria

Grids containing non-serial ultrathin sections (90 nm thick) were examined at a magnification of 6000× to count the total number of mitochondria per cell and mitochondrial size; or 8000× or 10,000× to count the number of immune-gold particles. Several grids were observed in order to obtain a total number of at least 30 (in A172 cells) or 50 (in U87MG cells) for each experimental group. Starting from the grid square corner, the whole of the sectioned pellet within that grid square was scanned in equally spaced parallel sweeps across the specimens. Each count was repeated at least 2 times by two blind observers. Mitochondria are characterized by a double-membrane containing matrix and a system of cristae. Although the basic morphology of the mitochondria is well stated, many variations can occur during physiological and functional activities as well as in pathological states. Altered mitochondria were defined according to ultrastructural criteria, being validated in previous studies as follows: (i) decreased electron density of the matrix (dilution, vacuolization, and presence of electron-lucent foci); (ii) fragmented, disorganized and ballooned cristae (intracristal swelling); (iii) partial or complete separation of the outer and inner membranes; (iv) presence of dense osmiophilic laminated or whorled membranes in mitochondria; (v) mitochondrial swelling [61,63]. 

Measurements of the mitochondria electron density and maximum and minimum diameter were carried out with ImageJ software 1.52v. In particular, in order to translate the mitochondria electron density into statistical values we analyzed digital micrographs at 6000×. For each micrograph, the mitochondrial electron density was normalized by calculating the ratio between the raw electron density of mitochondria and the mean cytoplasm raw electron density. The maximum and the minimum diameter of each mitochondrion were selected using the straight-line selection tool of ImageJ software and reported as micrometer.

### 4.5. Light Microscopy for MitoTracker Dye

MitoTracker-Green (MTR-G) dye Thermo-Fisher Scientific, Waltham, MA, USA) was used to stain healthy and unhealthy mitochondria in live cells [68,69].

For MTR-G dye, 5 × 10^2^ U87MG and A172 cells were grown in 24 well plates containing 1 mL/well of culture medium. At the end of the experiment the medium of each sample was removed and cells were incubated with a solution of green-MTR dye at 250 nM in culture medium serum free for 45 min at 37 °C and 5% CO_2_. At the end of incubation, MTR-G solution was removed and fresh pre-warmed medium was added. The stained cells were observed with a fluorescence microscope (Leica Microsystems, Wetzlar, Germany) and the optical density was analyzed using ImageJ software. 

### 4.6. Light Microscopy for LC3

For light microscopy, 5 × 10^4^ GBM cells were grown on poly-lysine slides placed in 24 well plates containing 1 mL/well of culture medium. Cells were washed in PBS and fixed with 4% paraformaldehyde in PBS for 15 min. Antigen retrieval was carried out in 0.1% TritonX-100 for 15 min in PBS and then blocked in PBS + 10% normal goat serum for 1 h at 21 °C. Cells were then incubated overnight at 4 °C in 1% normal goat serum in PBS containing anti-LC3 antibody diluted 1:50 (Table 2). After extensive washes in PBS, cells were incubated at 21 °C for 1 h with fluorophore-conjugated secondary antibody Alexa Fluor 488 (anti-rabbit, 1:200; Life Technologies, Carlsbad, CA, U.S.A.). After washing in PBS, slices were gently pulled out and transferred onto a coverslip and mounted with the mounting medium Fluorosheild (Sigma Aldrich). Slices were observed using the Nikon Eclipse 80i light microscope equipped with a fluorescent lamp and a digital camera connected to the NIS Elements Software for image analysis (Nikon, Tokyo, Japan).

### 4.7. SDS-PAGE Immune-Blotting

The GBM cell pellet was placed in an Eppendorf tube containing 20 μL of ice-cold lysis buffer with phosphatase and protease inhibitors to be homogenized. An aliquot of the homogenate was used for Bradford protein assay. Proteins (25 μg) were separated on SDS-polyacrylamide gels (Mini Protean TGX precast gel 4–20% gradient, Biorad, Milan, Italy) and transferred on nitrocellulose membranes (for mixed molecular weight; 75 V-75 min). Membranes were blocked for 2 h in Tween-20 Tris-buffered saline (TTBS) (100 mM Tris-HCl, 0.9% NaCl, 1% Tween 20, pH 7.4) containing 5% non-fat dry milk (Biorad). We used the following primary antibodies: i) mouse anti- phospho-p70 S6 kinase (1:1000; Cell Signaling, Danvers, MA, USA); ii) rabbit anti-β-actin (1:3000; Abcam, Cambridge, UK) as an internal standard for semi-quantitative protein measurement (Table 2). The anti-mouse phospho-p70 S6 kinase antibody binds two isoforms: p70 S6 and p85 S6 kinase. p85 S6 kinase is derived from the same gene and it is identical to p70 S6 kinase except for 23 extra residues at the amino terminus.

We performed immune-blotting analysis using two methods as follows:

(i) membranes were incubated overnight at 4 °C with anti-phospho-p70 S6 kinase together with anti-β-actin antibodies in TTBS containing 2.5% non-fat dry milk. Then, membrane was washed 3 times with TTBS buffer and incubated for 1 h with secondary peroxidase-coupled antibodies, anti-mouse and anti-rabbit (1:3000; Calbiochem, Milan, Italy). This approach allowed visualization of the bands from phospho-p70 S6 kinase protein and from the internal standard in the very same image. Bands were visualized with enhanced chemiluminescence reagents (Biorad, Milan, Italy).

(ii) one membrane was incubated overnight at 4 °C with primary mouse anti-phospho-p70 S6 kinase antibody in TTBS containing 2.5% non-fat dry milk, and then it was washed in TTBS and incubated for 1 h with peroxidase-labeled secondary antibody. After visualization with enhanced chemiluminescence, the membrane was stripped with a solution of distilled water and 3.5% acetic acid in the presence of 1% NaCl 5 M for 20 min, washed in TTBS, blocked in TTBS with 5% non-fat dry milk, and then incubated with primary rabbit anti-β-actin antibody (4 °C overnight) and peroxidase-labeled secondary antibody (1 h). Bands were visualized with enhanced chemiluminescence reagents (Biorad, Milan, Italy). Image analysis was carried out by ChemiDoc System (Bio-Rad Laboratories, Milan, Italy). Optical density was normalized for relative β-actin using ImageJ software.

### 4.8. Statistical Analyses

For MitoTracker the optical density of each sample was calculated. Values were expressed as the percentage of the fluorescent densitometry of each sample with respect to the Control. 

For qReal Time-PCR experiments statistical analyses using one-way analysis of variance (ANOVA) followed by the Bonferroni test using GraphPad Prism version 6.0 was used to test differences in gene expression in basal and treated cells (*p* ≤ 0.05). 

Inferential statistics to compare groups was carried out by using One-way analysis of variance, ANOVA, with Sheffè’s post-hoc analysis (H_0_, null hypothesis, was rejected for *p* ≤ 0.05).

For ultrastructural morphometry, values were expressed using the number as follows: (i) number of mitochondria per cell, (ii) number of mitochondria per cell positive for NRF2 or BNIP3, (iii) total number per cell of NRF2, LC3 and BNIP3 immune-gold particles, (iv) number of unstained vacuoles per cell, (v) number of LC3-positive vacuoles per cell, (vi) number of Cathepsin D positive vacuoles per cell, (vii) number of Cathepsin D + LC3 positive vacuoles per cells. Moreover, values for ultrastructural morphometry were also expressed as a ratio as follows: (i) the number of NRF2 immune-gold particles within mitochondria out of the number of cytoplasmic NRF2 immune-gold particles, (ii) the number of LC3 immune-gold particles within vacuoles out of the number of cytoplasmic LC3 immune-gold particles, (iii) the number of BNIP3 immune-gold particles within mitochondria out of the number of cytoplasmic BNIP3 immune-gold particles. The optical density of mitochondria was expressed as a percentage. Finally, we calculated the maximum or minimum diameter of mitochondria for each experimental group.

Values were reported as the mean or the mean percentage ± S.E.M. per cell from 50 cells (U87MG) or 30 cells (A172) per group in all counts. The maximum or minimum diameter were expressed as the mean ± S.E.M. from 250 mitochondria (U87MG) or 50 mitochondria (A172) per group, while the optical density of mitochondria and the area of mitochondria was expressed as the mean ± S.E.M. from 100 mitochondria (U87MG) and 50 mitochondria (A172) per group. 

The number of immune-fluorescent puncta for LC3 was expressed as the mean per cell in each experimental group (each group being representative of a specific dose and timing of rapamycin/vehicle saline in each cell line). Comparisons were made with ANOVA and for inferential statistics we applied the Scheffe’s post-hoc test. Differences between the various groups were considered to be significant when the null hypothesis H_0_ was estimated to be less than 5%.

For SDS-PAGE immune-blotting analysis, optical density was normalized for relative β-actin using ImageJ software. Data were expressed as the mean ± S.E.M. calculated from three blots and compared by using one-way ANOVA followed with Fisher post-hoc analysis (H_0_, null hypothesis, was rejected for *p* ≤ 0.05).

## Figures and Tables

**Table 1:** Primers used for quantitative Real Time PCR.

Target Gene	Target Sequence Number	Primer Sequences
*PGC1* *α*	NM_001330752	FW 5′- GAGCTTCTCAAATATCTGAC -3′ RW 5′- GGTCAGAGGAAGAGATAAA -3′
*TFAM*	NM_003201.3	FW 5′- AAGTCAGATTATGTCTTTGG -3′ RW 5′-TTCCAAGCAGTGTTAACTC-3′
*NRF2*	NM_006164.5	FW 5′- ACCATTTCAGATGAAACTTCAG -3′ RW 5′- AAACCACCCAATGCAGGAC -3′
*ND1*	LC178901.1	FW 5′- ATACCCATGGCCAACCTCCTACTCCTC -3′ RW 5′- GGTATGGGGAGGGGGGTTCATAG -3′,
*ND2*	LC178901.1	FW 5′- CCCTTACCACGCTACTCCTA -3′ RW 5′- GGCGGGAGAAGTAGATTGAA -3′
*ND4*	LC178901.1	FW 5′-AAAACTAATCGTCCCAACAATT -3′ RW 5′- ATAAGTGGCGTTGGCTTGCCATGATTG -3′
*ATP6*	LC178901.1	FW 5′ - CCTTCCACAAGGAACTCCAA -3′ RW 5′- GGTAGCTGTTGGTGGGCTAA -3′
*Cyt b*	LC178901.1	FW 5′- ATTCCTTCATGTCGGGACGAG -3′ RW 5′- GGGATGGCTGATAGGAGGTT -3′
*LC3*	NM_032514.4	FW 5′ -CATGAGCGAGTTGGTCAAGA-3′ RW 5′ -CCATGCTGTGCTGGTTCA- 3′
*Beclin1*	NM_001313998.2	FW 5′ -GGATGGTGTCTCTCGCAGAT- 3′ RW 5′ -TTGGCACTTTCTGTGGACAT- 3′
*β–Actin*	NM_001101.3	FW 5′-GTGCGTGACATTAAGGAG-3′ RW 5′-GCCAGACAGCACTGTGT-3′
*β-Globin*	NM_000518.4	FW 5′-CTAAGGTGAAGGCTCATG-3′ RW 5′- GATAGGCAGCCTGCACT-3

**Table 2:** Sources and identification codes for each antibody used in the present study.

Antibody	Purchaser	Cat#	Research Resource Identifier (RRID)
Rabbit anti-NRF2	Abcam	ab137550	AB_2687540
Rabbit anti-LC3	Abcam	ab128025	AB_11143008
Rabbit anti-BNIP3	Thermo Fisher Scientific	701696	AB_2607981
Mouse anti-Cathepsin-D	Sigma-Aldrich	C0715	AB_258707
EM Goat anti-Rabbit IgG, gold conjugate antibody	BBInternational	EM.GAR 10	AB_1769128
EM Goat anti-Rabbit IgG, gold conjugate antibody	BBInternational	EM.GAR 20	AB_1769136
Goat Anti-Mouse IgG (H+L)	BBInternational	EM.GMHL20/0.25	AB_1769164
Mouse anti-Phospho-p70 S6 Kinase	Cell Signaling	9025	AB_2734746
Alexa Fluor 488	Thermo Fisher Scientific	A-11008	AB_143165
Goat anti-Mouse IgG, H+L chain specific peroxidase conjugate antibody	Millipore	AP308P	AB_11215796
Goat anti-Rabbit IgG, H+L chain specific peroxidase conjugate antibody	Millipore	AP307P	AB_92641
Rabbit anti-beta Actin antibody	Abcam	ab34731	AB_722539

## Supplementary Materials

Supplementary materials can be found at https://www.mdpi.com/1422-0067/21/13/4570/s1.
