# Supplementary material for: Quantitative Ultrastructural Morphometry and Gene Expression of mTOR-Related Mitochondriogenesis within Glioblastoma Cells
Source: Int J Mol Sci. 2020 Jun 27;21(13):4570. doi: 10.3390/ijms21134570 (PMC7370179; doi:10.3390/ijms21134570)
Supplement: Supplementary file 1 [file ijms-21-04570-s001.pdf]

SUPPLEMENTARY MATERIAL

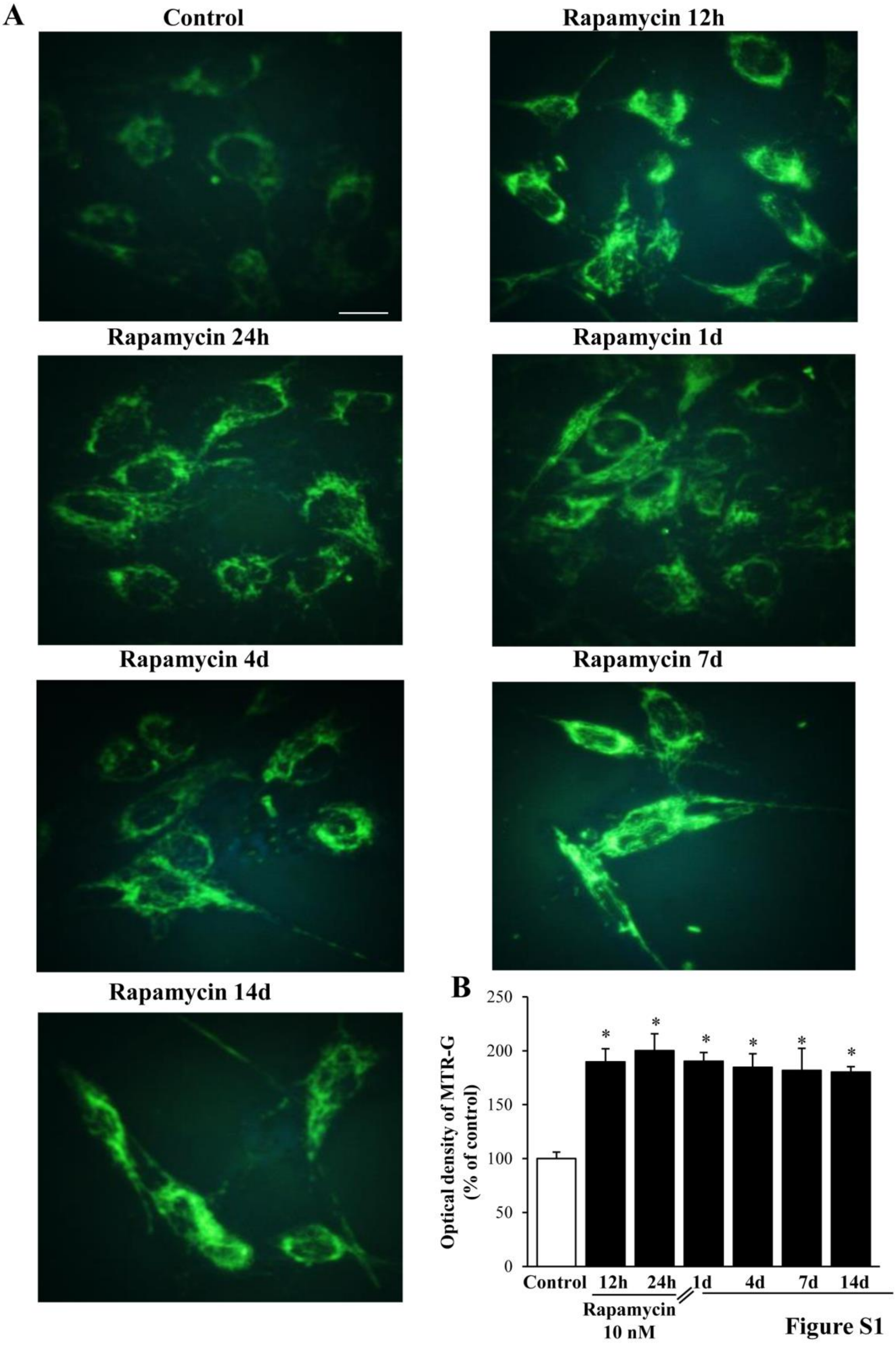

Figure S1. Rapamycin increases MitoTraker-green (MTR-G) in A172 cell line. (**A**) Representative fluorescent microscopy of A172 cells stained with 250 nM MTR-G dye. (**B**) Graph reports fluorescence at different time intervals. Values are the mean percentage±S.E.M. from 40 cells per group. *p ≤ 0.05 vs Control. Scale bar=25 μm.

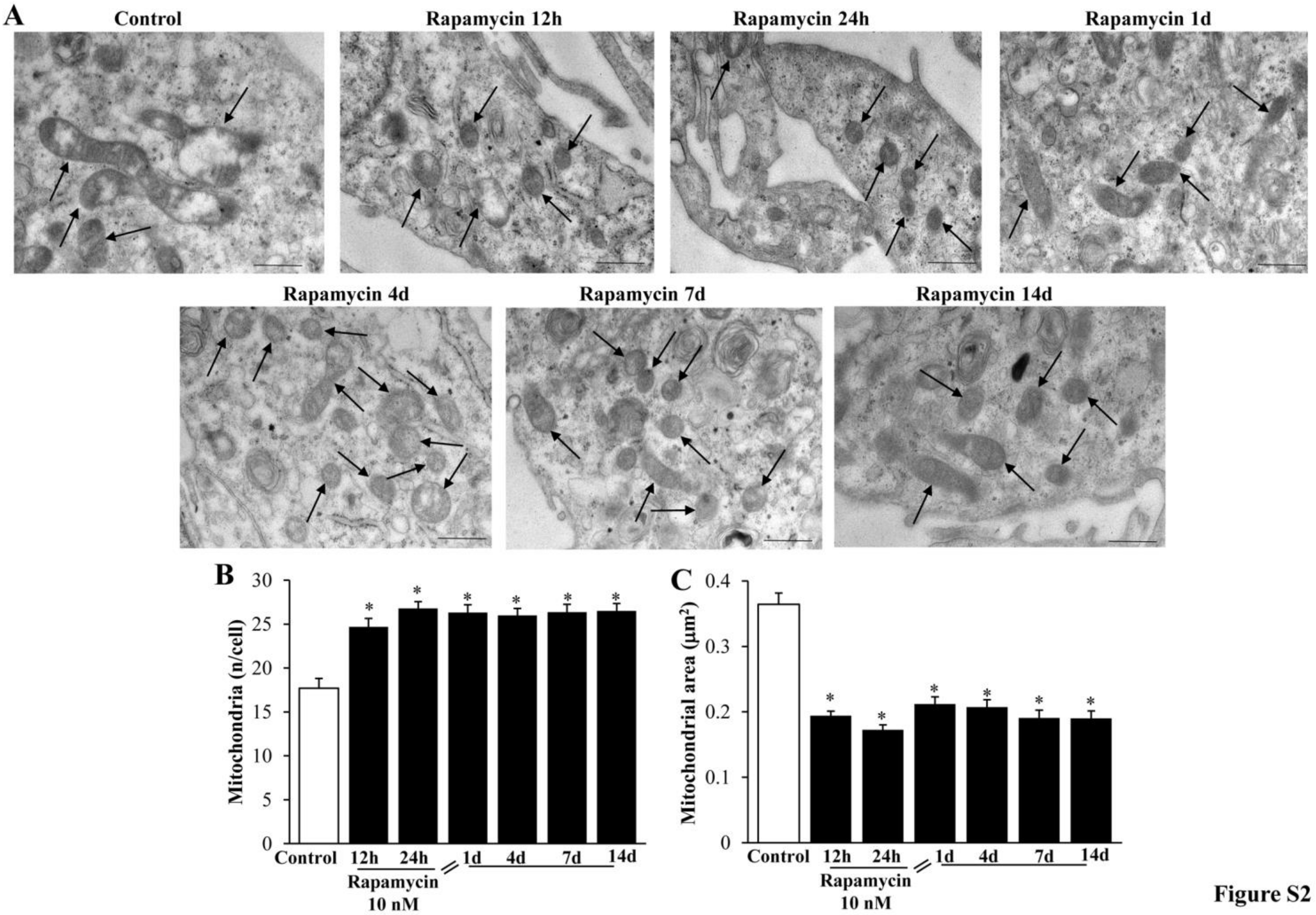

Figure S2. Rapamycin modifies mitochondrial number, area, and phenotype in A172 cell line. (**A**) Representative TEM micrographs showing mitochondria (black arrows) at different time intervals following 10 nM rapamycin. The graph (**B**) reports the number of mitochondria, which are increased at each time interval following rapamycin administration. Graph (**C**) measures the mitochondrial area, which is steadily and persistently decreased following rapamycin. This indicates that rapamycin produces an augment of mitochondria, which are much smaller in size. Values are the mean±S.E.M. from 30 cells per group, while mitochondrial area was calculated from 50 mitochondria per group. *$p \leq 0.05$ vs Control. Scale bar= 0.3 μm.

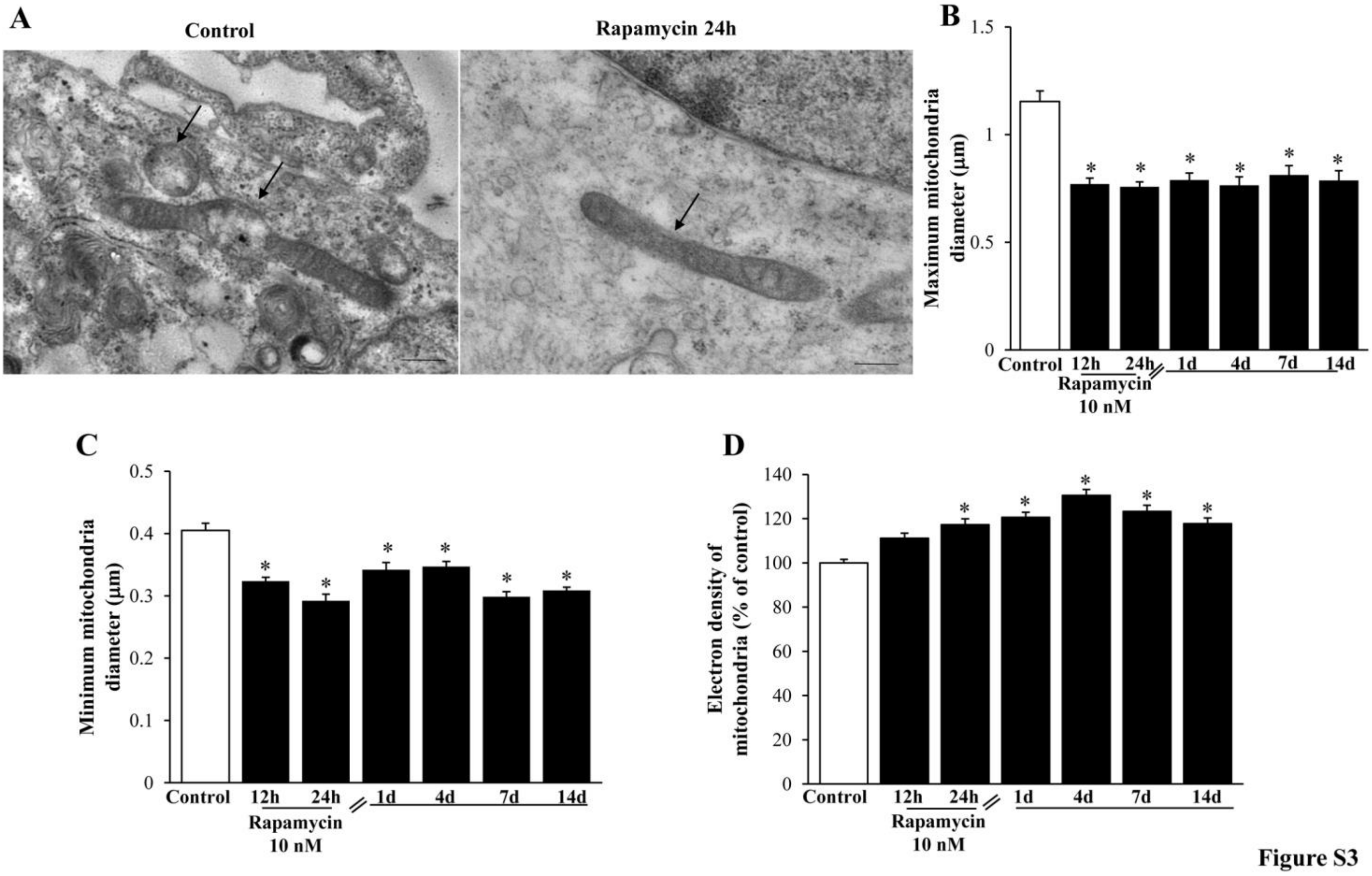

Figure S3

Figure S3. Rapamycin re-shapes mitochondria at different time intervals in A172 cell line. (**A**) Representative TEM micrographs showing mitochondria (black arrows) from Control and following 10 nM rapamycin at 24h. Graphs (**B**, **C**, **D**) confirm what reported in Figure 8. In fact, both the maximum (**B**) and minimum (**C**) mitochondrial diameter were decreased, whereas the mitochondrial electron density (**D**), which is expressed as a percentage of mitochondrial electron-density measured from Control, was increased. This increase was less pronounced compared with what measured in U87MG cell lines. Values were obtained from 50 mitochondria per group. *p ≤ 0.05 vs Control. Scale bar=0.2 μm.

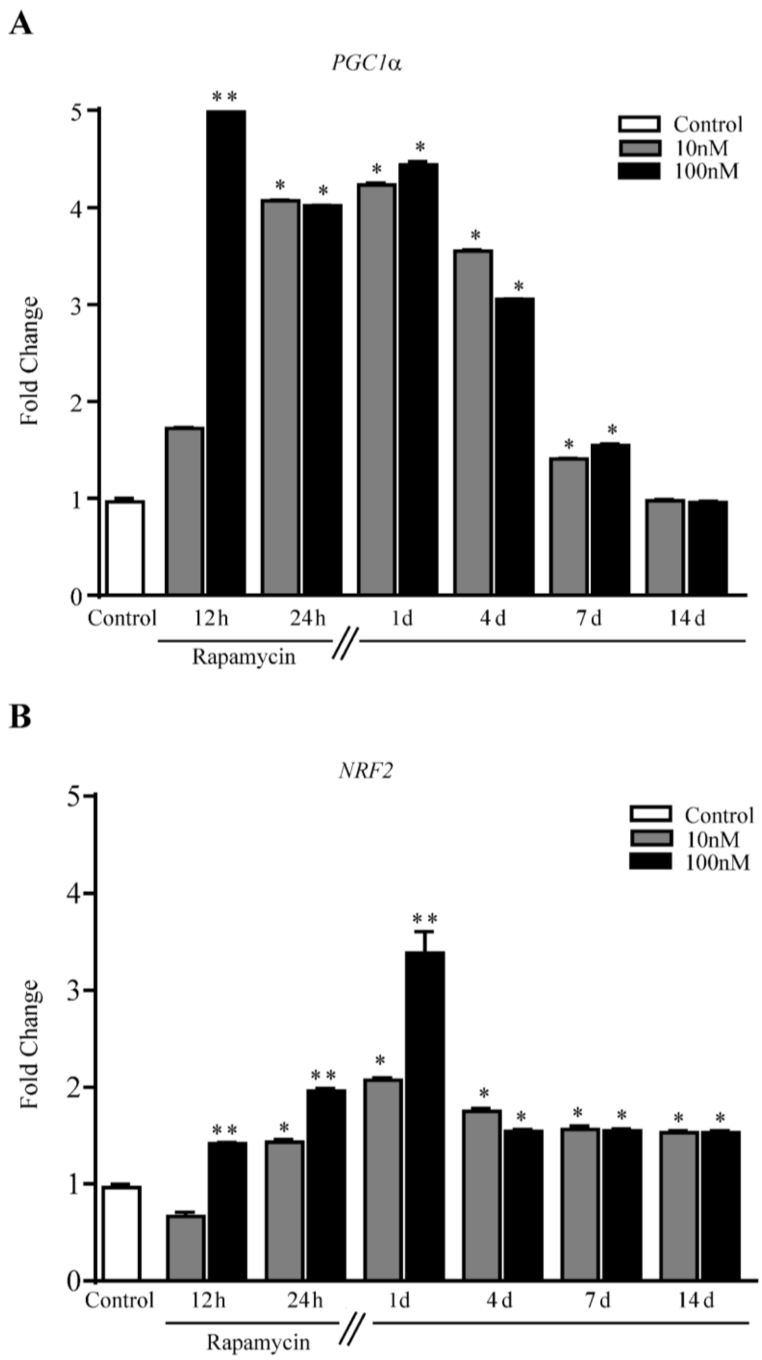

Figure S4

Figure S4. Rapamycin increases mitochondriogenesis-related genes in A172 cells. RT-PCR for (**A**) PGC1α, (**B**) NRF2. *p ≤ 0.05 vs Control; **p ≤ 0.05 vs Control and 10 nM rapamycin. Each RT-PCR was performed in triplicate and confirmed in two independent experiments using both beta-globin and beta-actin as internal references.

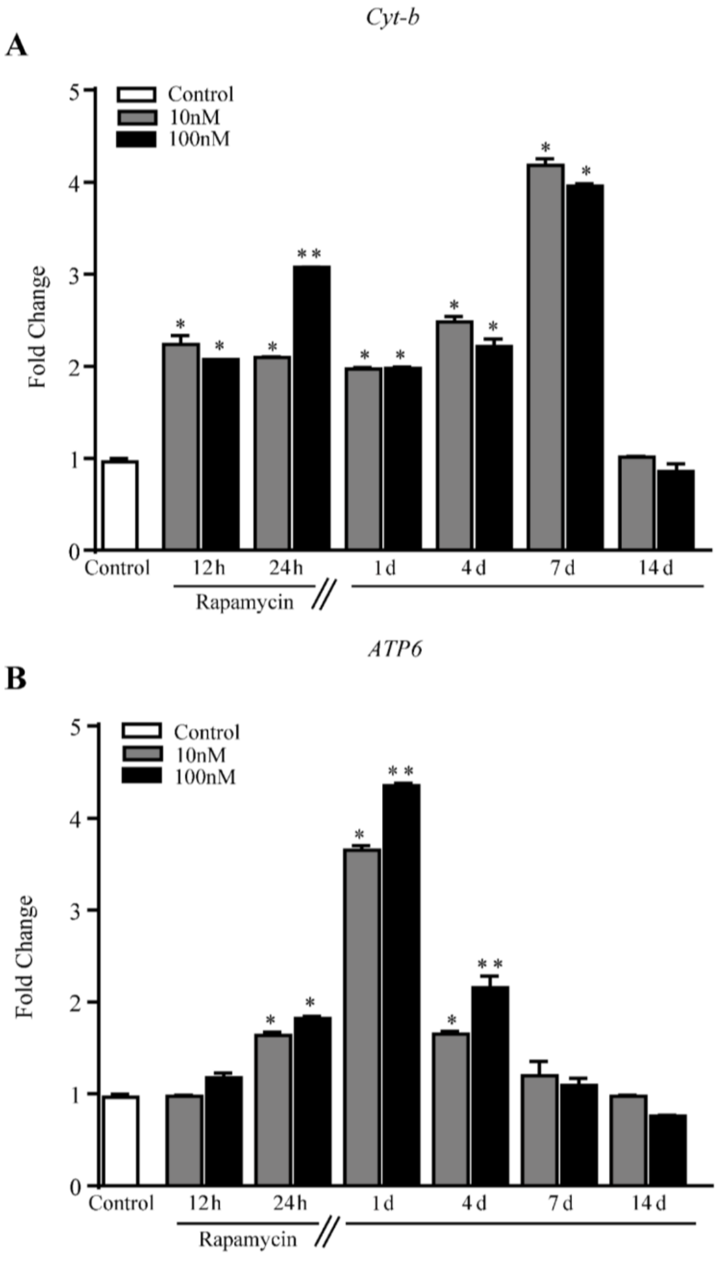

Figure S5

Figure S5. Rapamycin increases mRNA levels for constitutive mitochondrial genes in A172 cells. RT-PCR for (**A**) *Cyt-b,* and (**B**) *ATP6*. *p ≤ 0.05 vs Control. **p ≤ 0.05 vs Control and 10 nM rapamycin. Each RT-PCR was performed in triplicate and confirmed in two independent experiments using both beta-globin and beta-actin as internal references.

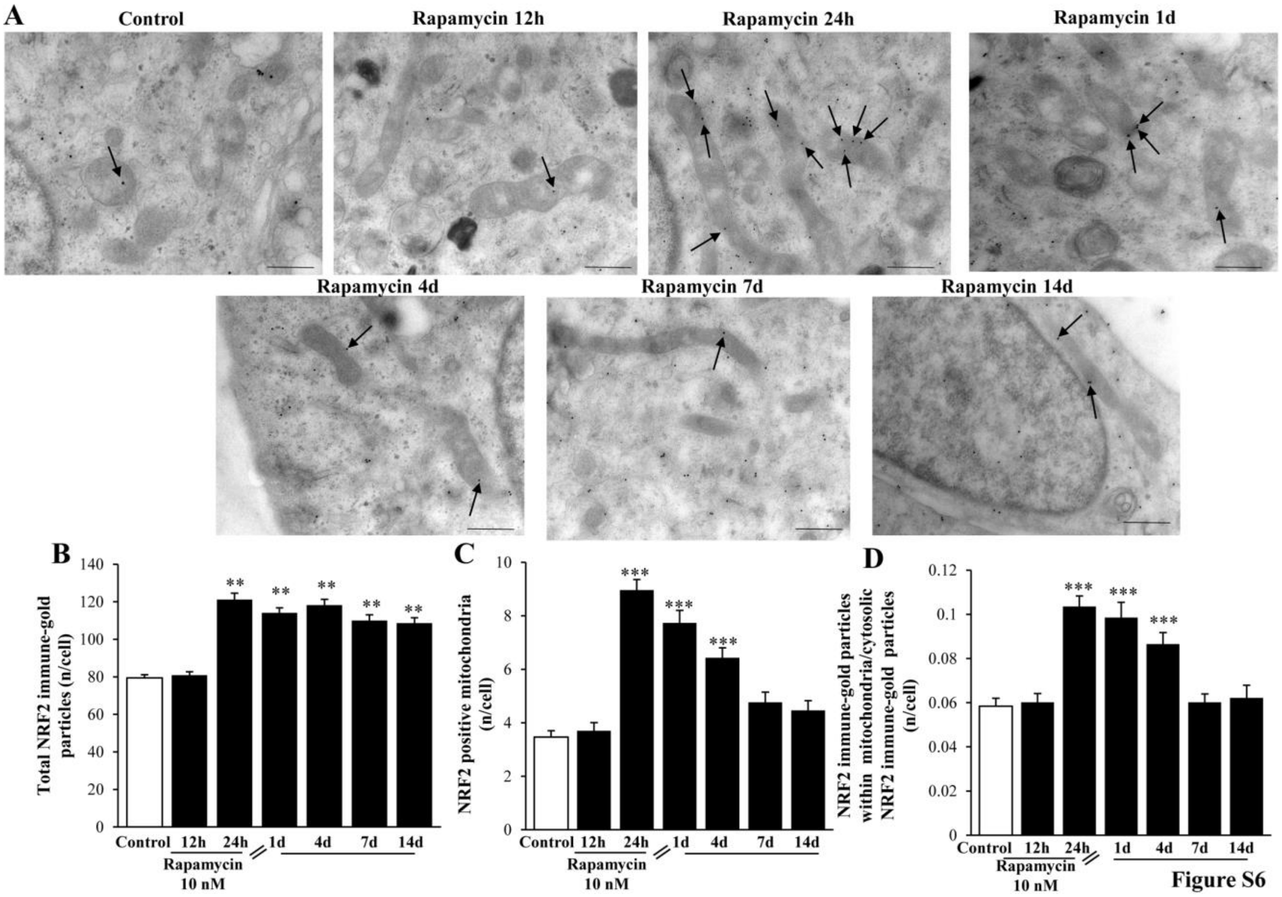

Figure S6. Rapamycin increases the amount of NRF2 immune-gold within A172 cells. (**A**) Representative TEM micrographs showing NRF2 immune-gold particles (black arrows) within mitochondria from A172 cells at different time intervals following rapamycin withdrawal. Graphs report the amount of NRF2 particles per cell (**B**), NRF2-positive mitochondria (**C**), and the ratio of mitochondrial to cytosolic NRF2 particles (**D**). **p ≤ 0 05 vs Control and 12h, ***p ≤ 0 05 vs other groups. Scale bar=0.25 μm.

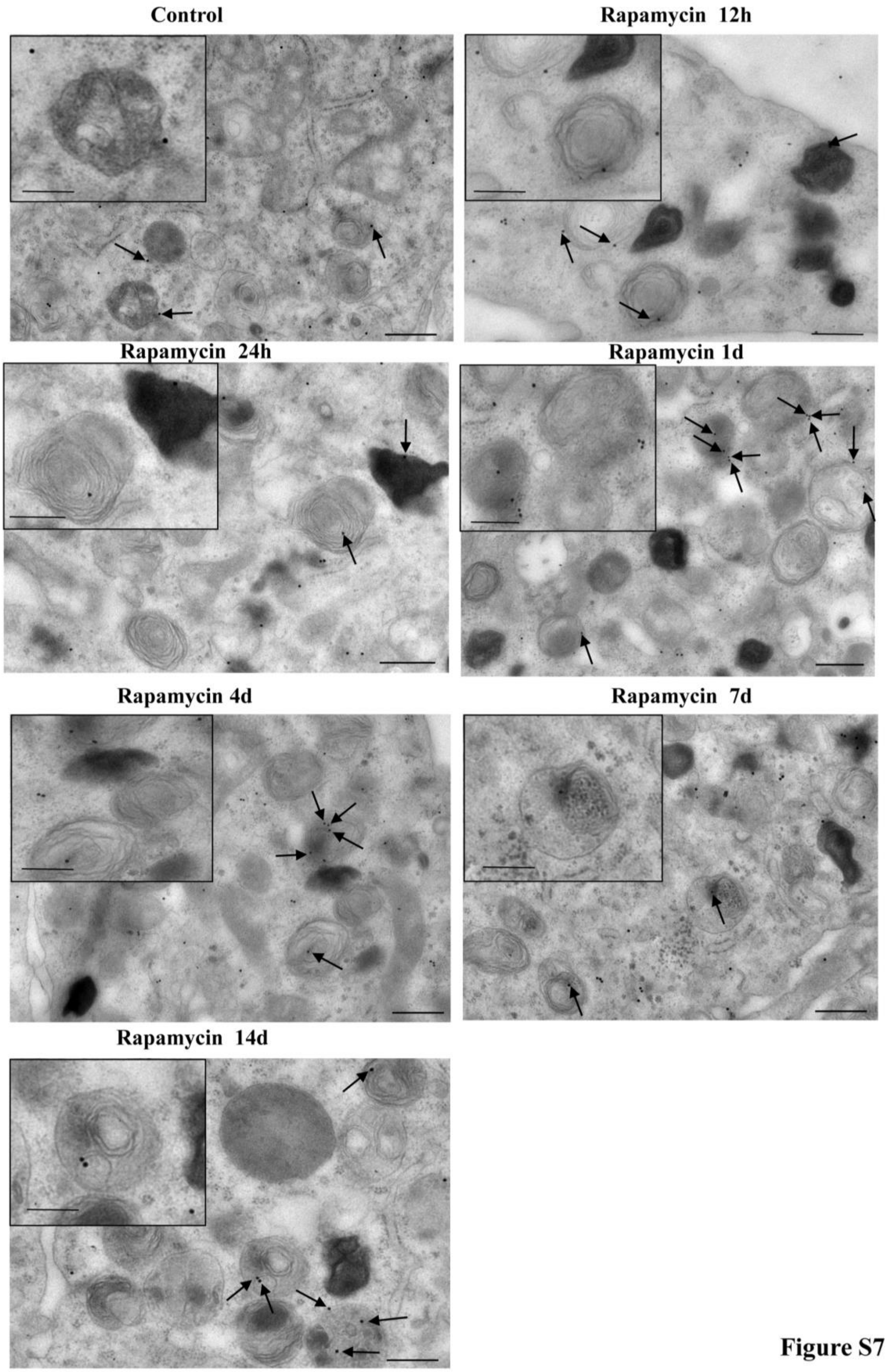

Figure S7. Rapamycin promotes the formation of LC3-stained and non-stained vacuoles in A172 cells. Representative pictures of A172 cells at different time intervals showing vacuoles in the cytoplasm. Inserts (black boxes) at high magnification show LC3-stained vacuoles. Black arrows point to LC3 immune-gold particles within vacuoles. Scale bar: 0.40 μm (low magnification); 0.25 μm (high magnification).

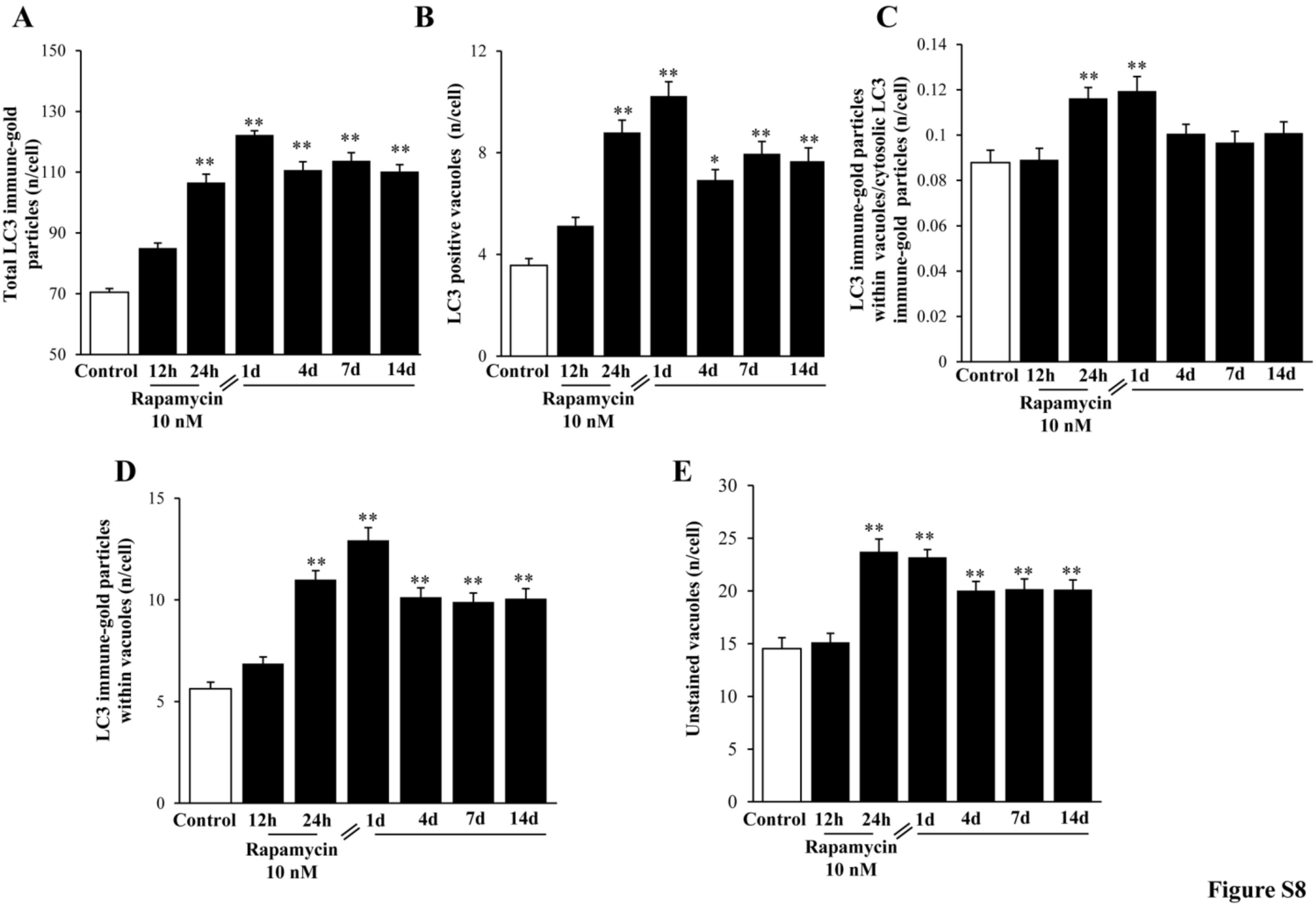

Figure S8

Figure S8. Rapamycin increases the amount of both LC3-stained and non-stained vacuoles in A172 cells. Graphs report the increase in LC3 immune-gold particles within cytosol (**A**), the LC3 positive-vacuole (**B**), the ratio of vacuolar to cytosolic LC3 (**C**) and LC3 immune gold particles within vacuoles (**D**). Rapamycin increased the vacuolar compartment also considering those vacuoles which did not stain for LC3 (**E**). *p ≤ 0.05 vs Control; **p ≤ 0.05 vs Control and 12h.

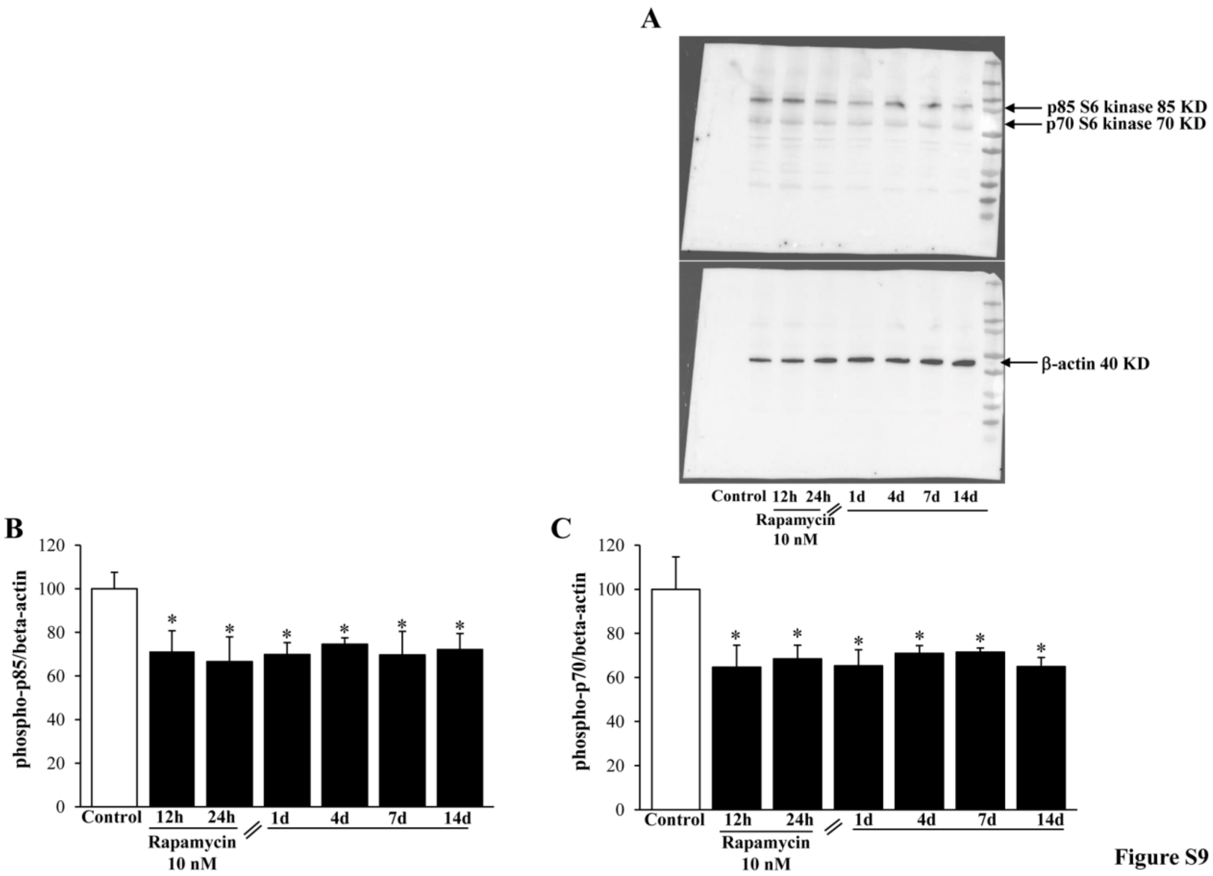

Figure S9. Modulation of mTORC1 downstream products phopspho-p85 S6K and phopspho-p70 S6K. (**A**) Representative SDS-PAGE immunoblotting of phopspho-p85 S6K and phopspho-p70 S6K (before membrane stripping) and β-actin (after membrane stripping) in Control and Rapamycin-treated U87MG cells. Rapamycin was administered continuously for 12h and 24h and it was withdrawn for 1d, 4d, 7d, and 14 d following 24h continuous exposure. (**B, C**) Graphs report a decrease in phopspho-p85 and phopspho-p70 S6K levels following rapamycin treatment. *p ≤ 0.05 vs Control. Densitometry was calculated by ImageJ software from three blots from three independent experiments.

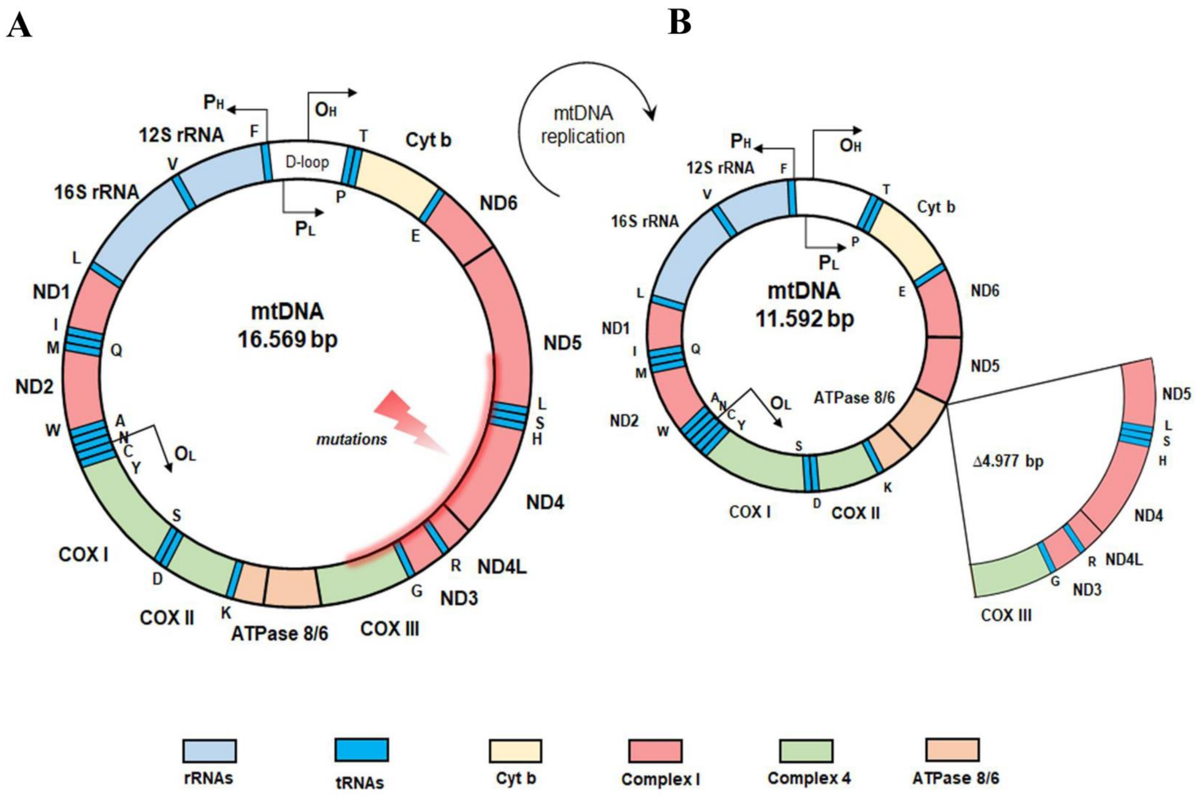

Figure S10

Figure S10. Representative cartoon of mtDNA gene re-arrangements leading to *ND4* deletion. (**A**): human mtDNA (16.569 bp); letter codes indicate tRNAs' cognate amino acids. The mtDNA locus which is mostly vulnerable to mtDNA mutations and re-arrangements is highlighted in red. (**B**): being placed in the most vulnerable region to DNA deletions, *ND4* is most likely to be lost during mtDNA replication [59,60].
